# ERα/SIRT1 signaling mediates the metabolic regulatory benefits of pterostilbene

**DOI:** 10.1016/j.jbc.2026.113471

**Published:** 2026-08-24

**Authors:** Tengteng Huang, Xiaoling Chen, Daiwen Chen, Jingdong Yin, Bing Yu, Hui Yan, Ping Zheng, Zhiqing Huang

**Affiliations:** 1 Key Laboratory for Animal Disease-Resistance Nutrition of China Ministry of Education, Institute of Animal Nutrition, Sichuan Agricultural University, Chengdu, Sichuan, P. R. China; 2 State Key Laboratory of Animal Nutrition and Feeding, College of Animal Science and Technology, China Agricultural University, Beijing, P. R. China

**Keywords:** pterostilbene, metabolic regulator, muscle fiber type, obesity, ERα, SIRT1

## Abstract

Pterostilbene (PTS), a more bioavailable analog of resveratrol (RES), is a promising natural metabolic regulator for improving metabolic health. The metabolic regulatory benefits of RES are believed to be associated with SIRT1 activation. However, the direct activation of SIRT1 by RES was proven to be an *in vitro* artifact. Here, we demonstrate that PTS is a more potent metabolic regulator than RES. More significantly, we have identified estrogen receptor α (ERα) as the intermediate signaling mediating SIRT1 expression by both RES and PTS. First, RES and PTS function as ERα agonists to stimulate *Sirt1* transcription. Second, RES and PTS act as stabilizers of ERα-SIRT1 interaction, blocking ubiquitination and thereby enhancing their protein stability. Third, RES and PTS promote ERα deacetylation, subsequently increasing ERα transactivation. Finally, skeletal muscle-specific ERα knockout attenuates the metabolic regulatory benefits of PTS. Together, our study demonstrates that PTS is a more potent metabolic regulator than RES by activating the ERα/SIRT1 signaling.

As a result of the lack of physical exercise and excessive consumption of high-fat and high-sugar diets among many individuals, obesity and metabolic diseases are highly prevalent worldwide in modern society (1). To address this issue, some metabolic regulators have been developed to enhance metabolic health (2). Among these, incretin-based therapies have achieved remarkable clinical success. For example, semaglutide, marketed as Wegovy for chronic weight management and Ozempic for type 2 diabetes, has demonstrated substantial efficacy in reducing body weight, improving glycemic control, and alleviating diabetic kidney disease in patients with type 2 diabetes. (3, 4). Several muscle-targeted metabolic regulator, such as synthetic drugs like AICAR, GW501516, and GSK4716, have been engineered (2). However, concerns about their long-term application, including potential side effects, high cost, and the need for sustained treatment, highlight the importance of developing safe, sustainable, and nutrition-derived metabolic regulators. As an alternative strategy, natural products with metabolic regulatory benefits may serve as long-term intervention as both medicinal and edible resources due to their milder effects and lower toxicity (5).

Resveratrol (RES, *trans*-3,5,4′-trihydroxystilbene) is a representative and extensively studied natural metabolic regulator. In animal studies, RES supplementation has demonstrated physiological effects analogous to those induced by exercise, including increased NAD^+^ production, activation of adenosine monophosphate-activated protein kinase (AMPK) and Sirtuin (SIRT), enhanced mitochondrial function and oxidative phenotype in skeletal muscle (6), improved endurance performance (7), and mitigation of obesity and metabolic disorders (8). However, its metabolic regulatory benefits in human clinical trials has been suboptimal, which may be attributed to its low bioavailability (9, 10). Pterostilbene (PTS, *trans*-3,5-dimethoxy-4′-hydroxystilbene), a dimethyl ether analog of RES, exhibits superior bioavailability and pharmacological activity compared to RES due to its enhanced hepatic metabolic stability and cell membrane permeability (11). Similar to RES, recent studies have shown that PTS promotes the oxidative phenotype of skeletal muscle, enhances exercise endurance, alleviates obesity, and exerts anti-diabetic effects, suggesting that it may serve as a more potent metabolic regulator than RES (12, 13). However, systematic comparisons between RES and PTS as metabolic regulators are lacking.

Furthermore, while RES and its analogs were initially proposed to exert metabolic benefits by activating SIRT1, their role as direct SIRT1 activators has been strongly challenged (14). It was reported that RES significantly enhanced SIRT1 activity in assays using an 7-amino-4-methylcoumarin (AMC) fluorophore labeled acetylated p53 peptide and demonstrated potential anti-aging effects, sparking widespread research interest in RES and its analog as SIRT1 activators (15). However, multiple independent studies have substantially challenged that the SIRT1-activating effect of RES depends on the presence of an AMC fluorophore conjugated to the substrate peptide (16, 17, 18), revealing that the direct activation of SIRT1 by RES was an *in vitro* artifact.

Sufficient evidence has demonstrated that RES supplementation indeed enhances both SIRT1 gene/protein expression and enzymatic activity (19). Consequently, recent investigations have focused on identifying the direct targets of RES that serve as intermediate signaling factors for SIRT1 activation. Several potential mechanisms have been elucidated, with one predominant hypothesis proposing that RES primarily activates AMPK–potentially through inhibition of cAMP phosphodiesterases (20) or mitochondrial ATP synthase (21, 22) –thereby inducing NAD^+^ biosynthesis and subsequent SIRT1 activation. However, studies employing low-dose RES treatment observed enhanced SIRT1 activity without significant AMPK activation (23). Furthermore, RES-mediated AMPK phosphorylation was shown to depend on SIRT1 (6). These findings imply the existence of AMPK-independent pathways for SIRT1 activation by RES. Another study revealed that RES promotes the interaction between SIRT1 and Lamin A, with Lamin A acting as a SIRT1 coactivator to enhance its activity (23). However, this study could not fully explain the effects of RES on SIRT1 gene/protein expression. Therefore, the mechanisms underlying RES-mediated upregulation of SIRT1 expression and activity remain to be fully elucidated.

Here, we demonstrate that PTS is a more potent metabolic regulator than RES. More significantly, we have innovatively identified estrogen receptor α (ERα) as a potential intermediate signaling factor that mediates SIRT1 expression and activation induced by both RES and PTS. RES and PTS function not only as direct ERα agonists to stimulate *Sirt1* transcription but also as stabilizers of the ERα-SIRT1 interaction to promote the protein stability of both ERα and SIRT1. In addition, skeletal muscle-specific ERα knockout attenuates the metabolic regulatory benefits of PTS in HFD-fed mice. Our study unravels the mechanism of how RES and its analogs promote SIRT1 expression and highlights the therapeutic potential of PTS as a substitute for RES and a target of the ERα-SIRT1 axis to achieve metabolic regulatory benefits.

## Results

### RES and PTS confer metabolic regulatory benefits under HFD and standard diet

**Figure 1:**
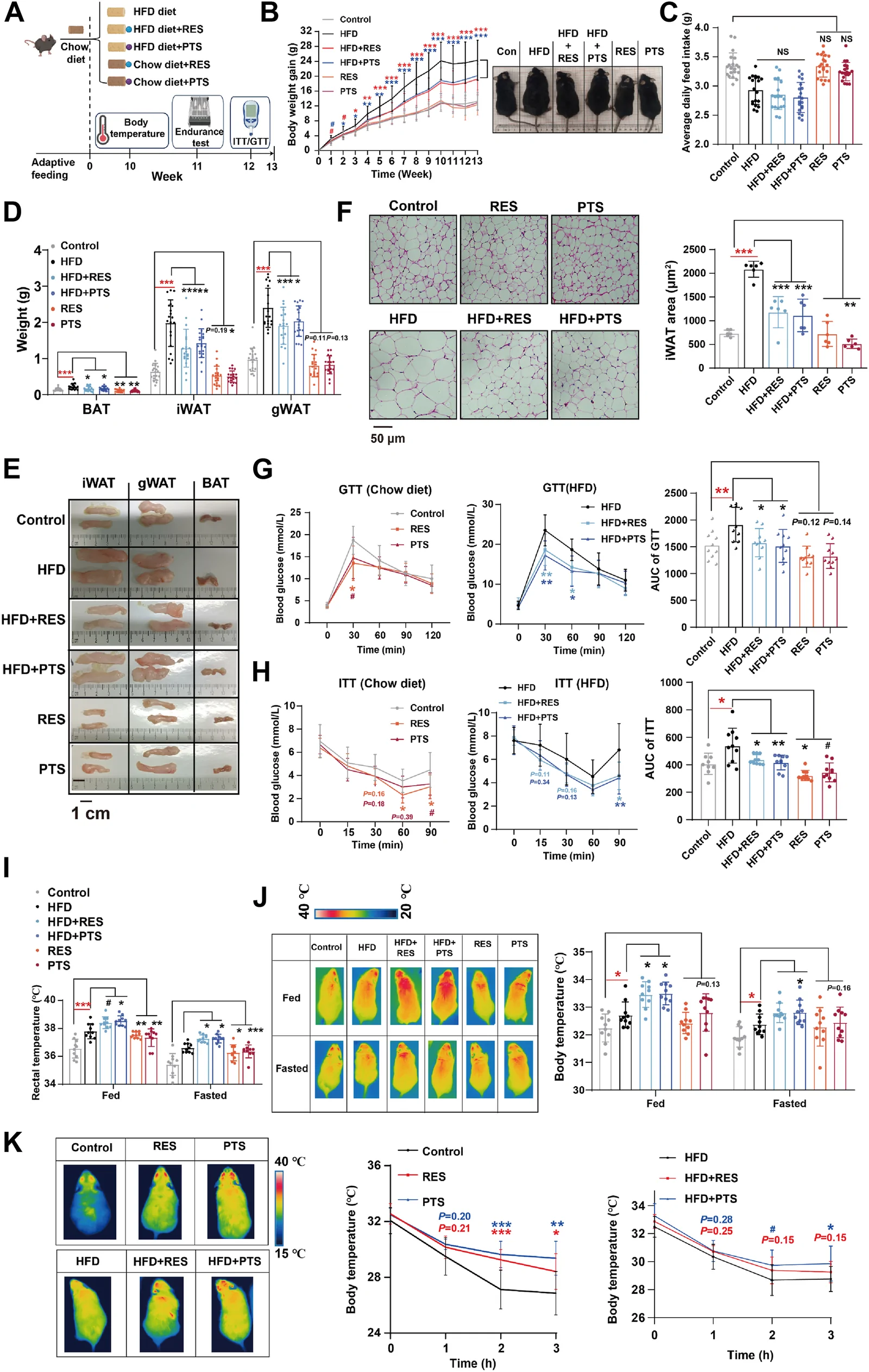
**RES and PTS alleviate obesity and metabolic disorders in mice under standard diet and HFD.***A*, experimental design schematic. Male mice were randomly divided into six groups (n = 20 per group): Chow diet (Control), HFD, HFD + 400 mg/kg RES (HFD + RES), HFD + 400 mg/kg PTS (HFD + PTS), chow diet + 400 mg/kg RES (RES), and chow diet + 400 mg/kg PTS (PTS). The experiment lasted 13 weeks. Body temperature was measured at week 10, endurance tests at week 11, and insulin tolerance test (ITT) and glucose tolerance test (GTT) at week 12. *B*, body weight gain curves during the 13-weeks experimental period and representative photographs of mice at the end of the experiment selected based on average body weight. (*C*) Average daily food intake. *D* and *E*, weights of BAT, iWAT, and gWAT (*D*), along with representative images (*E*). *F*, H&E staining of iWAT and adipocyte area quantification. *G* and *H*, blood glucose levels at different time points and corresponding area under the curve (AUC) in GTT (*G*) and ITT (*H*). *I* and *J*, rRectal temperature (*I*) and surface body temperature (*J*) under fed/fasted conditions. *K*, surface body temperature during cold exposure. For (*B–D*), n = 20 per group. For (*F*), n = 6 per group. For (*G–K*), n = 10 per group. One-way ANOVA followed by Duncan’s multiple range test for HFD groups (HFD, HFD + RES, HFD + PTS) and chow diet groups (Control, RES, PTS). #*p* < 0.1, ∗*p* < 0.05, ∗∗*p* < 0.01, and ∗∗∗*p* < 0.001. For line graphs, statistical significance markers in different colors correspond to comparisons between respective groups and the control or HFD group. Data are presented as means ± SD.

To systematically compare the metabolic regulatory benefits of dietary RES and PTS, mice fed HFD or standard diet received RES or PTS (400 mg/kg diet) for 13 weeks (Fig. 1*A*). RES and PTS alleviated HFD-induced obesity without affecting body weight in mice fed a standard diet (Fig. 1*B*), although they did not change daily food intake (Fig. 1*C*). Consistent with the reduction in body weight, RES and PTS decreased the weight of BAT, iWAT, and gWAT under HFD (Fig. 1*D*). Notably, under a standard diet, both RES and PTS reduced BAT weight, while PTS decreased iWAT weight (Fig. 1*D*). Representative images of adipose tissues corroborated these findings (Fig. 1*E*). H&E staining of iWAT revealed that both RES and PTS reduced adipocyte area under HFD, and only PTS reduced adipocyte area under standard diet (Fig. 1*F*). Next, we evaluated glucose and insulin tolerance. Both RES and PTS lowered blood glucose levels under HFD in GTT and ITT (Fig. 1, *G* and *H*). Additionally, in standard-diet-fed mice, RES and PTS lowered blood glucose levels in ITT (Fig. 1, *G* and *H*). To assess thermogenic effects, we measured rectal and surface body temperatures. RES and PTS increased rectal temperature under both fed and fasted conditions in mice fed either HFD or standard diet (Fig. 1*I*). In HFD-fed mice, RES and PTS increased surface temperature during fed states, while only PTS enhanced surface temperature during fasted states (Fig. 1*J*). During cold exposure, both RES and PTS enhanced surface temperature in standard-diet-fed mice starting at 2 h post-cold exposure, whereas only PTS improved surface temperature in HFD-fed mice after 2 h post-cold exposure (Fig. 1*K*). We also detected the expression of several lipid metabolism-related proteins in iWAT and mitochondrial complex protein expression in BAT. It was found that PTS suppressed the protein expression of the lipogenesis-related protein PPARγ under HFD (Fig. S1*A*). Both RES and PTS increased the protein expression of the lipolysis-related protein p-HSL under standard diet and HFD (Fig. S1*A*). Furthermore, RES and PTS differentially upregulated mitochondrial complex protein expression in BAT under standard diet and HFD (Fig. S1*B*).

We then investigated the effects of RES and PTS on the skeletal muscle oxidative phenotypes. Under both HFD and standard diet, RES and PTS improved endurance capacity (Fig. S2*A*), without altering skeletal muscle weight (Fig. S2*B*). Representative images of skeletal muscle revealed a distinct reddish coloration in RES- and PTS-treated groups, a morphological feature characteristic of slow muscle fibers (Fig. S2*B*). In TA and GAS muscle, RES and PTS promoted the shift from fast to slow muscle fiber under both HFD and standard diet (Fig. S2, *C* and *D*). In SOL muscle (predominantly composed of slow muscle fibers with high mitochondrial content), RES and PTS upregulated the mitochondrial complex protein expression (Fig. S2*E*) and alleviated mitochondrial ultrastructure (Fig. S2*F*). Together, these data demonstrate that at a dosage of 400 mg/kg diet, RES and PTS exhibit comparable metabolic regulatory benefits in promoting skeletal muscle oxidative phenotype and mitochondrial function, inducing thermogenesis, enhancing endurance capacity, mitigating obesity, and improving glucose homeostasis under HFD conditions. Moreover, even under a standard diet, RES and PTS still elicited modest metabolic regulatory effects beyond alleviating obesity.

### At low doses, PTS is a more effective metabolic regulator than RES in HFD-fed mice

**Figure 2:**
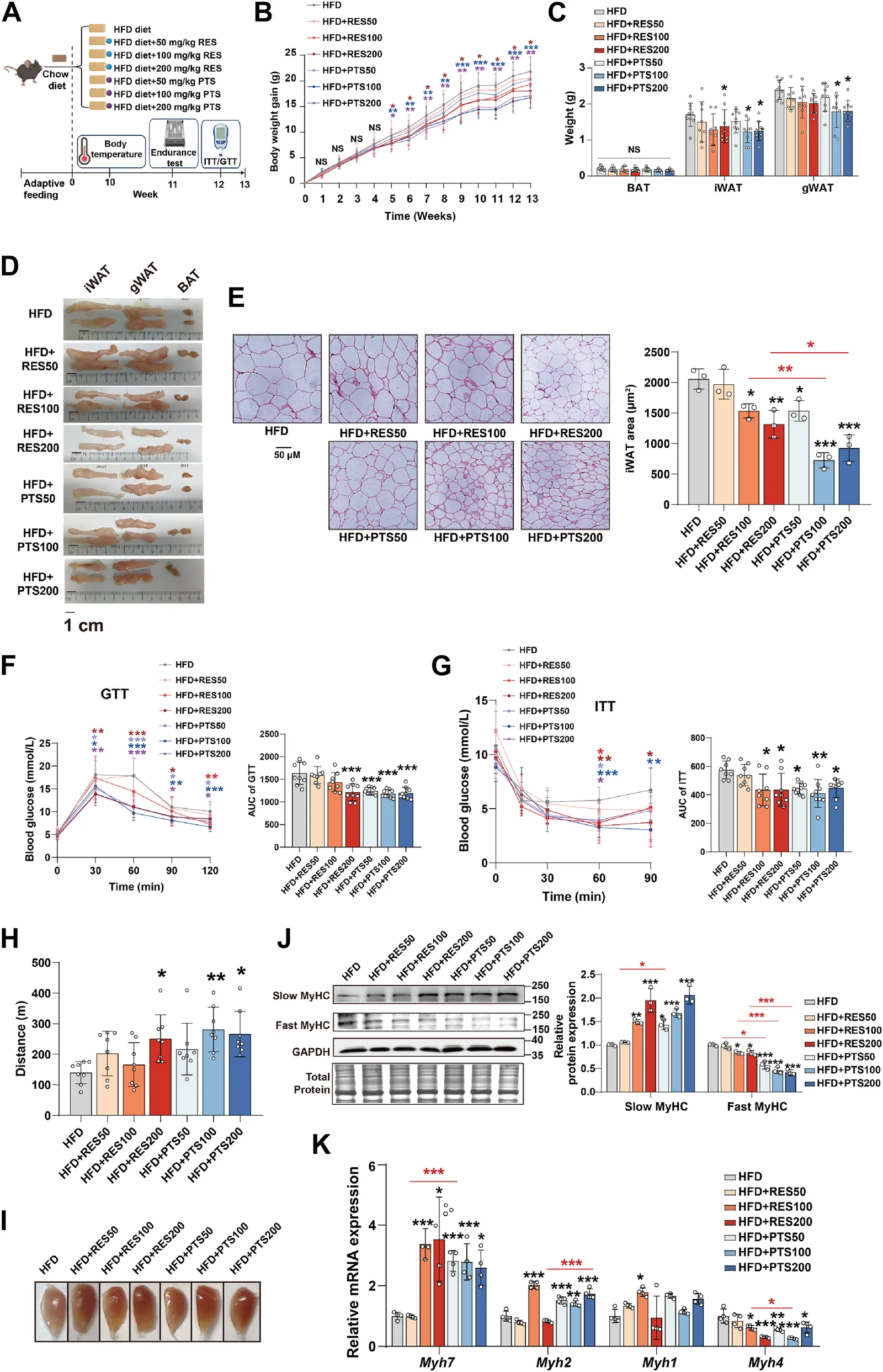
**Comparative dose-response effects of PTS and RES on skeletal muscle oxidative phenotype and metabolic disorders in HFD-fed mice.***A*, experimental design schematic. Male mice were randomly divided into seven groups (n = 8–9 per group): HFD, HFD+50 mg/kg RES (RES50), HFD + 100 mg/kg RES (RES100), HFD + 200 mg/kg RES (RES200), HFD + 50 mg/kg PTS (PTS50), HFD + 100 mg/kg PTS (PTS100), HFD + 200 mg/kg PTS (PTS200). The experiment lasted 13 weeks. Body temperature was measured at week 10, endurance tests at week 11, and insulin tolerance test (ITT) and glucose tolerance test (GTT) at week 12. *B*, body weight gain curves during the 13-weeks experimental period. (*C* and *D*) Weights of BAT, iWAT, and gWAT (*C*), along with representative images (*D*). (*E*) H&E staining of iWAT and adipocyte area quantification. *F* and *G*, blood glucose levels at different time points and corresponding area under the curve (AUC) in GTT (*F*) and ITT (*G*). *H*, running distance in endurance test. (*I*) Representative images of skeletal muscle morphology. (*J*) The protein expression of slow MyHC and fast MyHC in TA muscle. (*K*) The mRNA expression of Myh7, Myh2, Myh1, and Myh4 in TA muscle. For (*B*), (*C*), (*F*), (*G*), and (*I*), n = 8 to 9 per group. For (*E*) and (*J*), n = 3 per group. For (*K*), n = 4 per group. One-way ANOVA followed by Duncan’s multiple range test was applied for multiple-group comparisons. *Asterisks* directly above *bars* indicate significance compared to the control group. *Horizontal lines* with *asterisks* indicate direct comparisons between the connected groups. For line graphs, statistical significance markers in different colors correspond to comparisons between respective groups and HFD group, ∗*p* < 0.05, ∗∗*p* < 0.01, and ∗∗∗*p* < 0.001. Data are presented as means ± SD.

It was observed that RES and PTS exhibited comparable metabolic regulatory benefits when supplemented at 400 mg/kg diet, potentially due to they reaching saturation concentrations for eliciting such effects. This observation led us to hypothesize that PTS might exhibit superior efficacy to RES at lower concentrations. To test this hypothesis, we conducted a comparative evaluation of RES and PTS supplementation at different doses (50, 100, and 200 mg/kg diet) to assess their metabolic regulatory benefits in HFD-fed mice (Fig. 2*A*). RES alleviated HFD-induced weight gain at 200 mg/kg, whereas PTS exerted the effect at 100 to 200 mg/kg (Fig. 2*B*). Similarly, RES reduced iWAT weight at 200 mg/kg diet, while PTS reduced both iWAT and gWAT at 100 to 200 mg/kg (Fig. 2*C*). Representative images of adipose tissues corroborated these findings (Fig. 2*D*). In H&E staining of iWAT, RES reduced iWAT adipocyte area at 100 to 200 mg/kg, while PTS exerted the effect at 50 to 200 mg/kg (Fig. 2*E*). In the glucose homeostasis assay, RES lowered blood glucose at 200 mg/kg in GTT and 100 to 200 mg/kg in ITT, whereas PTS reduced blood glucose at 50 to 200 mg/kg in both GTT and ITT (Fig. 2, *F* and *G*). For skeletal muscle oxidative phenotype and endurance capacity, RES enhanced endurance at 200 mg/kg, whereas PTS enhanced endurance at 100 to 200 mg/kg (Fig. 2*H*). RES promoted fast-to-slow muscle fiber conversion at 100 to 200 mg/kg, whereas PTS exerted these effects at doses as low as 50 mg/kg (Fig. 2, *I*–*K*). In terms of thermogenesis, PTS exhibited superior effects to RES at lower doses, promoting baseline, fasting, and cold-exposure body temperatures (Fig. S3, *A* and *B*). In addition, PTS was more effective than RES in upregulating mitochondrial complex protein expression and mitochondrial copy number in BAT and SOL (Fig. S3, *C–F*). Moreover, both RES and PTS increased the expression of thermogenic and beige adipocyte-related genes, while PTS showed superior efficacy compared with RES (Fig. S3, *G* and *H*). Generally, RES required at least 200 mg/kg to alleviate HFD-induced obesity, whereas PTS was effective at 100 mg/kg. In addition, RES required at least 100 mg/kg to exhibit other metabolic regulatory effects, whereas PTS was effective at 50 mg/kg.

In C2C12 myotubes, PTS (effective at ≥ 0.5 μM) exhibited superior efficacy to RES (effective at ≥ 1 μM) in promoting the transition from fast to slow muscle fibers (Fig. S4, *A* and *B*). Furthermore, PTS demonstrated stronger effects than RES in upregulating the expression of mitochondrial transcription factor genes (Fig. S4*C*), mitochondrial complex genes (Fig. S4*D*), and mitochondrial complex proteins (Fig. S4*E*). Similarly, in 3T3-L1 cells, PTS more effectively suppressed the expression of the lipogenic protein FASN while enhancing the expression of the lipolytic protein p-HSL, compared to RES (Fig. S4*F*). Seahorse analysis demonstrated that RES and PTS promoted mitochondrial oxidative metabolism, with PTS showing a stronger enhancement of maximal respiration, ATP production, and spare respiratory capacity compared with RES (Fig. S5, *A–E*). In contrast, both compounds suppressed glycolytic metabolism, with PTS exhibiting a stronger inhibitory effect than RES, as indicated by greater reductions in ECAR, glycolysis, glycolytic capacity, and glycolytic reserve (Fig. S5, *F–I*). Together, our *in vitro* findings again demonstrate that PTS is a more potent metabolic regulator than RES.

### ERα is identified as the potential signaling mediator for SIRT1 activation by RES and PTS

To identify the potential signaling mediator for SIRT1 activation by RES and PTS, we constructed a library containing 24 natural and synthetic SIRT1 activators (Table S1). Potential targets for these SIRT1 activators, RES and PTS were predicted using PharmMapper (Fig. S6, *A* and *B*). These common targets (intersection size >5) of SIRT1 activators were further intersected with the top 50 predicted targets for RES and PTS (Fig. S6*C*). Finally, the resulting common targets, along with SIRT1, were submitted to perform PPI analysis (Fig. S6*D*). The results revealed that (1): ERα was a potential target for half of the SIRT1 activators (Fig. S6*A*) (2). ERα was the top predicted target for PTS and ranked second for RES (Fig. S6*B*) (3). PPI analysis revealed a potential interaction between ERα and SIRT1 (Fig. S6*D*). Furthermore, analysis of a mouse tissue single-cell database revealed that *Esr1* is predominantly expressed in skeletal muscle (especially in satellite cells) and adipose tissue (Fig. S7*A*). qPCR validation of mouse tissue expression profiles confirmed that *Esr1* is highly expressed in skeletal muscle and adipose tissue, especially in TA and gWAT (Fig. S7*B*). Based on these findings, we speculate that ERα might be the potential signaling mediator for SIRT1 activation in skeletal muscle and adipose tissue.

**Figure 3:**
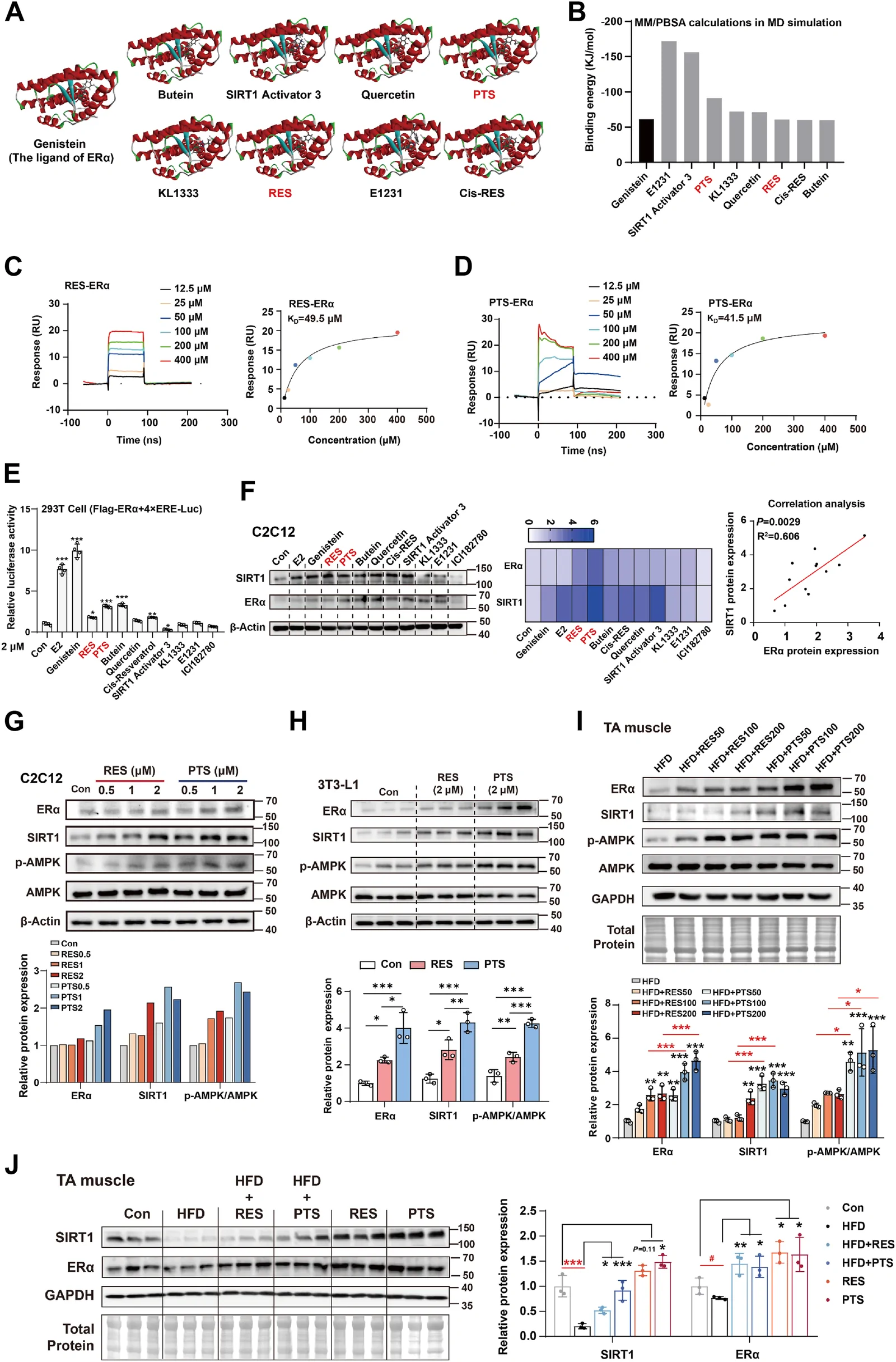
**ERα is identified as the potential signaling mediator for SIRT1 activation by RES and PTS.***A*, molecular docking of several SIRT1 activators and ERα, using the ERα activation site as the binding pocket. *B*, binding free energy calculated using the MM/PBSA method in molecular dynamics simulations. *C* and *D*, SPR detection of RES (*C*) or PTS (*D*) binding to ERα, showing response values and KD values. (*E*) 293T cells transfected with 4 × ERE-Luc and Flag-ERα plasmids were treated with small molecules (2 μM) for 1 day. The cells were then collected for luciferase assays. *F*, Differentiated C2C12 myotubes were treated with DMSO control or small molecules (2 μM) for 1 day, followed by protein extraction and Western blot analysis. *Left panel*: Immunoblot images. *Middle panel*: Heatmap depicting relative protein expression levels of ERα and SIRT1. *Right panel*: Correlation analysis of ERα and SIRT1 protein expression values, performed by linear regression with Pearson’s correlation coefficient (R2). *G* and *H*, differentiated C2C12 myotubes or 3T3-L1 cells were treated with DMSO control, RES or PTS for 1 day. The protein expression of ERα, SIRT1, AMPK, and p-AMPK in C2C12 myotubes (*G*) and 3T3L-1 cells (*H*). *I* and *J*, protein expression of ERα, SIRT1, AMPK, and p-AMPK in TA muscle. For (*E*), n = 4 per group. For (*H–J*), n = 3 per group. One-way ANOVA followed by Duncan’s multiple range test was performed for multiple-group comparisons. *Asterisks* directly above *bars* indicate significance compared to the control group. Horizontal lines with asterisks indicate direct comparisons between the connected groups. ∗*p* < 0.05, ∗∗*p* < 0.01, and ∗∗∗*p* < 0.001. Data are presented as means ± SD.

Molecular docking analysis of the above 13 SIRT1 activators using ERα as a potential target was performed, and 8 activators were predicted to bind to the ERα activation pocket (Fig. 3*A*). Further MD simulations of these 8 activators showed stable binding with ERα, as indicated by RMSD analysis (Fig. S8). Subsequent MM/PBSA binding energy calculations revealed that these compounds exhibited binding affinities comparable to or even stronger than genistein (Fig. 3*B*). SPR experiments further confirmed the binding affinity of RES and PTS to ERα, with K_D_ values of 49.5 μM and 41.5 μM, respectively (Fig. 3, *C* and *D*). Furthermore, SPR analysis showed that E2 directly interacted with ERα with a high binding affinity (K_D_ = 0.19 μM) (Fig. S9*A*). Competitive SPR assays further demonstrated that pterostilbene reduced the E2–ERα binding response (Fig. S9*C*). Moreover, in the presence of E2, pterostilbene failed to exhibit a concentration-dependent binding response (KD = 27.7 μM in the absence of E2), suggesting a potential overlap between the binding regions of pterostilbene and E2 on ERα (Fig. S9, *B* and *C*). Additionally, luciferase reporter assays showed that estradiol (E2), genistein and some natural SIRT1 activators (RES, PTS, butein, and cis-RES) possessed ERα activation activity. Specifically, E2 increased ERα reporter activity by 7.64-fold, whereas RES and PTS increased ERα activation by 1.76-fold and 3.11-fold, respectively, corresponding to approximately 23.0% and 40.7% of the activation level induced by E2 (Fig. 3*E*). Protein level analysis revealed that these SIRT1 activators promote the protein expression of both ERα and SIRT1, with a strong positive correlation between the protein levels of ERα and SIRT1 (R^2^ = 0.606) (Fig. 3*F*). In C2C12 myotubes (Fig. 3*G*), 3T3-L1 cells (Fig. 3*H*), and the TA muscle (Fig. 3*I*), both RES and PTS promoted ERα and SIRT1 protein expression, as well as AMPK phosphorylation, with PTS showing superior effects to RES. Furthermore, RES and PTS rescued the HFD-induced decline in ERα and SIRT1 levels in the TA muscle (Fig. 3*J*). Together, these findings suggest that certain natural SIRT1 activators, including RES and PTS, may function as ERα activators to promote ERα expression and activation.

### PTS promotes *Sirt1* transcription *via* ERα

**Figure 4:**
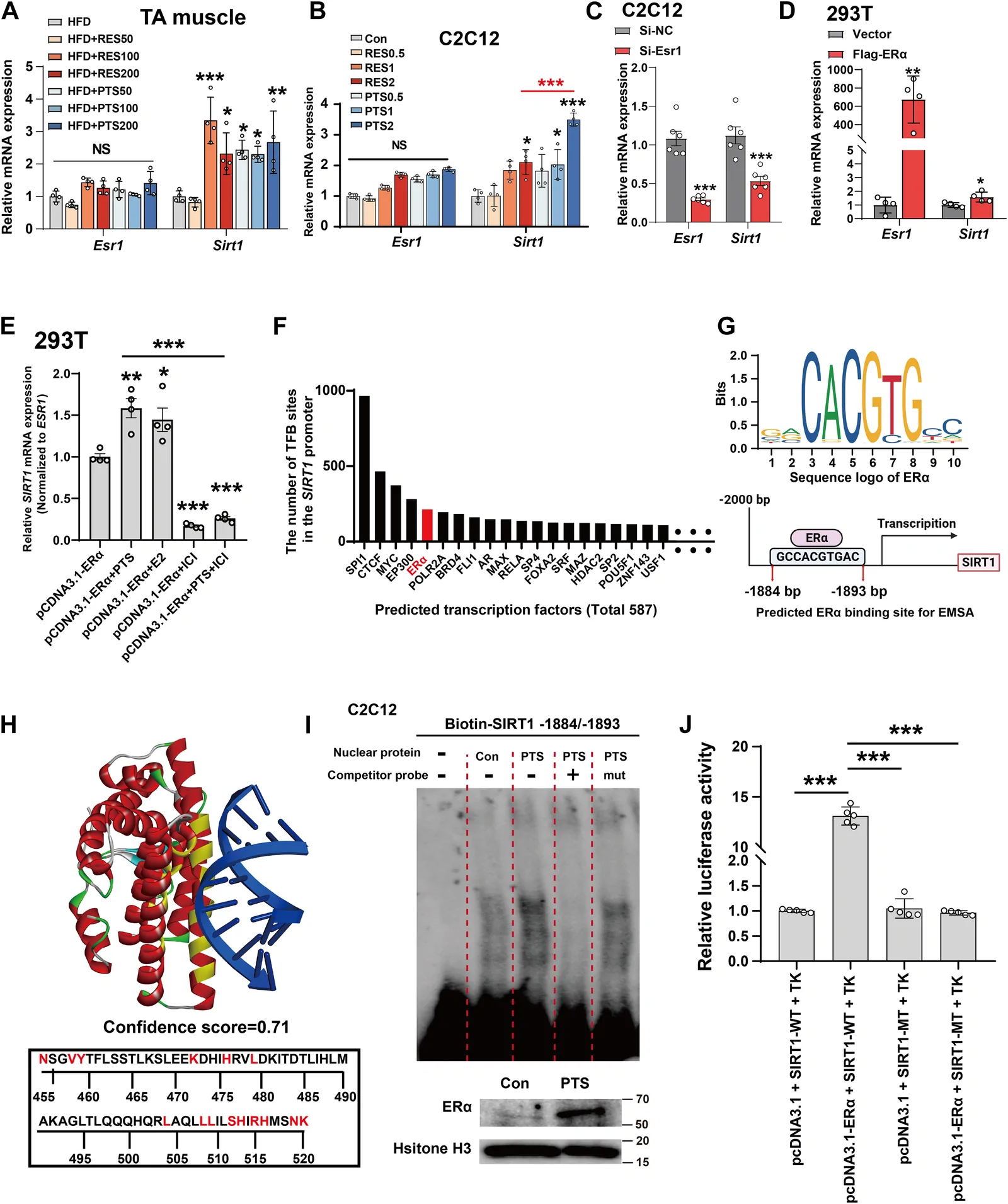
**PTS promotes *Sirt1* transcription *via* ERα.***A*, relative mRNA expression of Esr1 and Sirt1 in TA muscle. *B*, differentiated C2C12 myotubes were treated with DMSO control, 0.5 to 2 μM RES (RES0.5-RES2), or 0.5 to 2 μM PTS (PTS0.5-PTS2) for 1 day, followed by RNA extraction and qPCR analysis. Relative mRNA expression of *Esr1* and *Sirt1* in C2C12 myotubes. *C*, differentiated C2C12 myotubes were transfected with siRNA targeting Esr1 (Si-Esr1) for 1 day, followed by RNA extraction and qPCR analysis. Relative mRNA expression of *Esr1* and *Sirt1* in 293T cells. *D*, 293T cells transfected with vector or Flag-ERα plasmids for 1 day, followed by RNA extraction and qPCR analysis. Relative mRNA expression of *Esr1* and *Sirt1* in 293T cells. *E*, 293T cells transfected with Flag-ERα plasmids were treated with DMSO control, 2 μM PTS, 2 μM E2, 5 μM ICI (ERα antagonist), or 2 μM PTS +5 μM ICI for 1 day, followed by RNA extraction and qPCR analysis. Relative mRNA expression of SIRT1 in 293T cells. *F*, number of transcription factor binding sites in the Sirt1 promoter sequence (upstream 2 kb), predicted by AnimalTFDB. *G*, ERα motif sequence logo, and shared binding sites for ERα and the *Sirt1* promoter sequence (upstream 2 kb), predicted by AnimalTFDB, HIFtarget, and JASPAR. *H*, docking of ERα with the key *Sirt1* sequence using HDOCK (http://hdock.phys.hust.edu.cn/). Confidence scores above 0.7 indicate high binding probability, scores between 0.5 and 0.7 suggest possible binding, and scores below 0.5 indicate unlikely binding. The amino acids involved in the interaction are highlighted in *red*. *I*, differentiated C2C12 myotubes were treated with DMSO control or 2 μM PTS for 1 day, followed by nuclear protein extraction. Nuclear protein was used for EMSA. ERα protein expression in the nuclear extract is shown below. “Mut” refers to the mutated competitive probe. *J*, relative dual luciferase reporter gene activity, calculated as the ratio between firefly luciferase intensity and renilla luciferase intensity. For (*A*) and (*B*) and (D) and (*E*), n = 4 per group. For (*J*), n = 5 per group. For (*C*), n = 6 per group. One-way ANOVA followed by Duncan's multiple range test was performed for multiple-groups comparisons. Student’s *t* test was performed for two-groups comparisons. *Asterisks* directly above *bars* indicate significance compared to the control group. *Horizontal lines* with *asterisks* indicate direct comparisons between the connected groups. ∗*p* < 0.05, ∗∗*p* < 0.01, and ∗∗∗*p* < 0.001. Data are presented as means ± SD.

qPCR analysis revealed that RES and PTS did not affect *Esr1* gene expression but promoted *Sirt1* gene expression in the TA muscle (Fig. 4*A*) and C2C12 myotubes (Fig. 4*B*). Additionally, siRNA-mediated knockdown of *Esr1* reduced *Sirt1* gene expression (Fig. 4*C*), whereas overexpression of ERα in 293T cells increased *Sirt1* expression (Fig. 4*D*). Furthermore, treatment with PTS or E2 further enhanced Sirt1 expression in ERα-overexpressing cells, while the ERα antagonist ICI182780 abolished this effect (Fig. 4*E*). These data imply that ERα may act as a transcription factor to promote *Sirt1* transcription. Prediction of transcription factor binding sites in the *Sirt1* promoter region revealed numerous ERα binding sites (Fig. 4*F*). A high-confidence, common binding site sequence (−1884 bp to −1893 bp) from multiple prediction tools was selected for further validation (Fig. 4*G*). DNA-protein docking showed a high confidence score of binding between ERα and this promoter sequence (Fig. 4*H*). EMSA results (Fig. 4*I*) showed that the nuclear protein produced a shifted band (lane 2), which was further intensified by PTS treatment (lane 3). The band was abolished by the competitive probe (lane 4) but restored by the mutated probe (lane 5), confirming that ERα binds to this putative promoter sequence and that pterostilbene promotes this binding by enhancing ERα nuclear protein expression. Dual-luciferase reporter assays showed that overexpression of both SIRT1 and ERα reporter plasmids increased luciferase activity, while the effect was lost upon mutation of the SIRT1 reporter plasmid, confirming that ERα promote *Sirt1* transcription (Fig. 4*J*). Collectively, these results demonstrate that PTS promotes *Sirt1* transcription *via* ERα.

### PTS enhances protein stability of ERα and SIRT1 by promoting ERα-SIRT1 interaction

Based on the results from Figs. S5*D* and 3*F*, which suggest a potential interaction between ERα and SIRT1, we sought to further confirm this interaction and investigate whether RES or PTS could enhance it. According to the previously proposed MD stimulation method for predicting PPI stabilizers (24), an ideal PPI stabilizer usually possesses a dual-binding pattern, which includes a higher binding affinity to the protein-protein complex and a similar binding affinity to both proteins. Using this MD stimulation method, we predicted PPI stabilizer of Erα-SIRT1 interaction.

**Figure 5:**
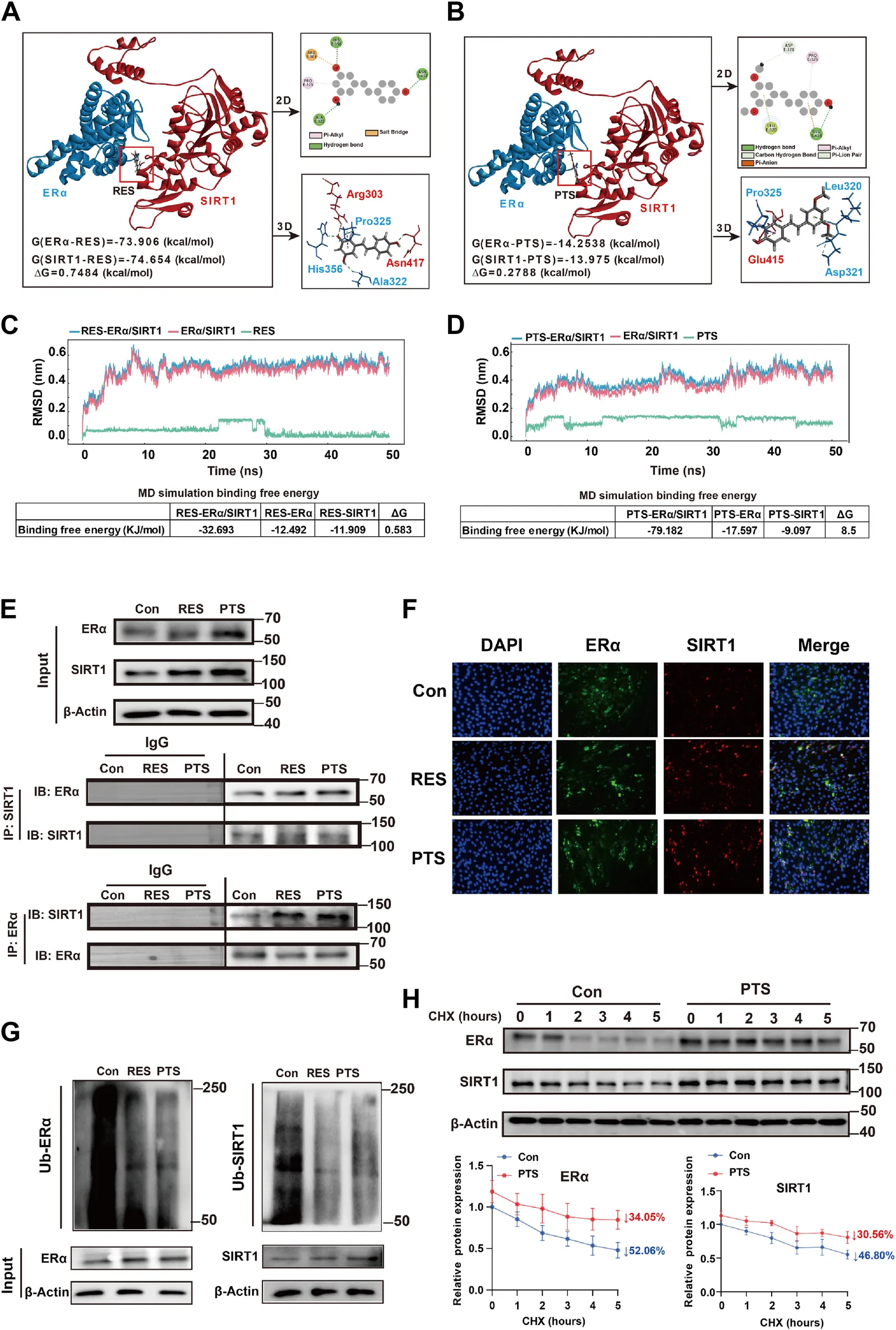
**RES and PTS enhance protein stability of ERα and SIRT1 by promoting ERα-SIRT1 interaction.***A* and *B*, molecular docking of RES (*A*) and PTS (*B*) with ERα-SIRT1. 3D binding mode diagrams and interacting amino acids in 2D binding mode along with binding free energies are shown. *C* and *D*, molecular dynamics (MD) simulation of RES (*C*) or PTS (*D*) with ERα-SIRT1. RMSD plots and binding free energies are displayed. *E–G*, differentiated C2C12 myotubes were treated with DMSO control, 2 μM RES or 2 μM PTS for 1 day. Co-IP analysis of ERα-SIRT1 interaction (*E*). Immunofluorescence staining showing ERα (*green*), SIRT1 (*red*) and nuclei (DAPI, *blue*) (*F*). Co-IP analysis was also performed to detect Ub-ERα and Ub-SIRT1 expression (*G*). *H*, differentiated C2C12 myotubes were treated with DMSO control or 2 μM PTS for 19 h, followed by treatment with CHX (50 μg/ml) + 2 μM PTS (or DMSO control) for 5 h. Proteins were collected every hour for 5 h, and western blotting was used to detect ERα and SIRT1 protein expression.

In the molecular docking analysis, RES and PTS both fit into the binding pockets formed at the interaction interface (Fig. 5, *A* and *B*). RES exhibited similar binding energies for both ERα (−73.906 kJ/mol) and SIRT1 (−74.654 kJ/mol) (Fig. 5*A*), while PTS showed similar binding energies for ERα (−14.254 kJ/mol) and SIRT1 (−13.975 kJ/mol) (Fig. 5*B*). In the MD simulations, the RMSD plots indicated that both RES and PTS gradually stabilized their binding to the ERα-SIRT1 contact surface-binding pockets, maintaining the dual-binding mechanism (Fig. 5, *C* and *D*). In addition, among the 13 SIRT1 activators previously investigated, cis-RES, RES, and PTS were found to most closely align with the dual-binding mechanism in MD simulations (Table S2).

These prediction results imply that RES and PTS may act as stabilizers of the ERα-SIRT1 interaction. Experimental validation confirmed this, as Co-IP assays revealed an interaction between ERα and SIRT1, and both RES and PTS enhanced this interaction (Fig. 5*E*). Additionally, ERα and SIRT1 exhibited predominant nuclear co-localization, with RES and PTS further intensifying this co-localization (Fig. 5*F*). The strengthened ERα-SIRT1 interaction led to a reduction in their ubiquitination (Fig. 5*G*), thereby increasing their stability (Fig. 5*H*).

Through analysis of the preferential interacting amino acids of RES/PTS-ERα-SIRT1 in molecular docking and free energy decomposition of the amino acids in MD simulations, we identified Proline325 (Pro325) of ERα as the key amino acid for stabilizing the ERα-SIRT1 interaction promoted by RES and PTS (Fig. S10, *A* and *B*). We further computationally mutated Pro325 to Ala325 of ERα (Fig. S10*C*), and MD simulations showed that after the mutation, the dual-binding pattern of RES or PTS with ERα-SIRT1 was weakened, as evidenced by an increase in ΔG (RES-ERα-SIRT1: from 0.583 to 5.78 kJ/mol, PTS-ERα-SIRT1: from 8.5 to 10.608 kJ/mol), and the strong binding affinity to the weaker stabilizer-binding partner was reduced (Fig. S10, *D* and *E*). We constructed a mutant ERα plasmid (mutERα, Pro325 to Ala325 of ERα), and subsequent experiments showed that after the mutation, the enhancement of Erα-SIRT1 interaction by RES and PTS was reduced (Fig. S10*F*), leading to increased ubiquitination of ERα and SIRT1 (Fig. S10*G*) and decreased their protein stability (Fig. S10*H*). In summary, the section demonstrates that RES and PTS promote the interaction between ERα and SIRT1, thereby preventing their ubiquitination and consequently enhancing their stability.

### PTS stabilizes ERα and SIRT1 by blocking MDM2-mediated ubiquitination

**Figure 6:**
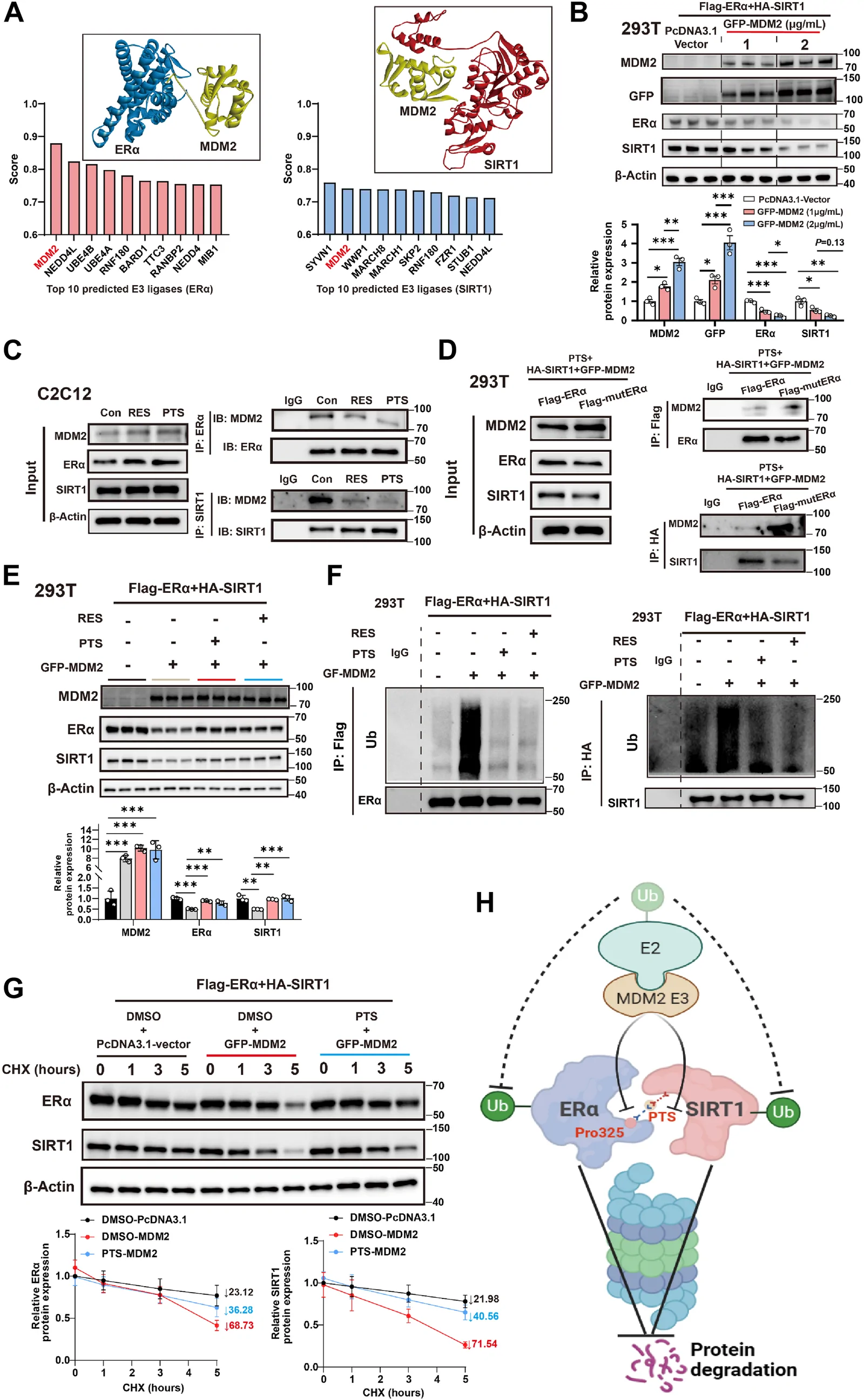
**PTS stabilizes ERα and SIRT1 by blocking MDM2-mediated ubiquitination.***A*, predicted E3 ubiquitin ligases for ERα (*Left*) or SIRT1 (*Right*) and protein docking results of ERα-MDM2 and SIRT1-MDM2 interaction. E3 ubiquitin ligases were predicted using Ubibrowser 3.0 (http://ubibrowser.bio-it.cn/ubibrowser_v3/), and protein docking was performed using ZDOCK. *B*, 293T cells transfected with vector or GFP-MDM2 were treated for 1 day. Protein expression of MDM2, GFP, ERα, and SIRT1. *C*, differentiated C2C12 myotubes were treated with DMSO control, 2 μM RES or 2 μM PTS for 1 day. Co-IP detection of ERα-MDM2 and SIRT1-MDM2 interaction. *D*, 293T cells transfected with GFP-MDM2, HA-SIRT1, and Flag-ERα (or Flag-mutERα, proline 325 to alanine substitution) were treated with 2 μM PTS for 1 day. Co-IP detection of ERα-MDM2 and SIRT1-MDM2 interaction. *E* and *F*, 293T cells transfected with GFP-MDM2 (or vector), HA-SIRT1, and Flag-ERα were treated with DMSO control, 2 μM RES or 2 μM PTS for 1 day. The protein expression of MDM2, ERα and SIRT1 (*E*). Co-IP detection of Ub-ERα and Ub-SIRT1 expression (*F*). *G*, 293T cells transfected with GFP-MDM2 (or vector), HA-SIRT1, and Flag-ERα were treated with DMSO control or 2 μM PTS for 19 h, followed by treatment with CHX (50 μg/ml) + 2 μM PTS (or DMSO control) for 5 h. The protein expression of ERα and SIRT1. *H*, schematic model illustrating how PTS stabilizes ERα and SIRT1 by inhibiting MDM2-mediated ubiquitination. For (*B*), (*E*), and (*G*), n = 3 per group. One-way ANOVA followed by Duncan’s multiple range test was performed for multiple-groups comparisons. ∗*p* < 0.05, ∗∗*p* < 0.01, and ∗∗∗*p* < 0.001. Data are presented as means ± SD.

To investigate the mechanism by which PTS prevents the ubiquitination-dependent degradation of ERα and SIRT1, we hypothesized that enhanced ERα-SIRT1 interaction might hinder the binding of an E3 ubiquitin ligase to either protein. By predicting potential E3 ligases for ERα or SIRT1, we found that MDM2 ranked highly and was a shared E3 ligase for both (Fig. 6*A*). Molecular docking and MD simulations revealed that MDM2 binds precisely to the regions of ERα or SIRT1 involved in their interaction (Figs. 6*A* and S11), suggesting that strengthening the ERα-SIRT1 interaction may block MDM2 binding. To validate this hypothesis experimentally, we overexpressed MDM2 in 293T cells and observed that ERα and SIRT1 protein levels decreased with increasing MDM2 transfection (Fig. 6*B*). Furthermore, Co-IP assays showed that RES and PTS reduced MDM2-ERα interaction and MDM2-SIRT1 interaction in C2C12 myotubes (Fig. 6*C*). Conversely, mutating key amino acids involved in PTS-enhanced ERα-SIRT1 binding promoted their interaction in 293T cells (Fig. 6*D*). Additionally, RES and PTS counteracted MDM2-induced reductions in ERα and SIRT1 protein levels (Fig. 6*E*), increases in ubiquitination (Fig. 6*F*), and decreases in protein stability (Fig. 6*G*). In summary, the section demonstrates that PTS enhances ERα-SIRT1 interaction, thereby obstructing the binding of the E3 ligase MDM2 and subsequently preventing ubiquitination-mediated degradation of ERα and SIRT1 (Fig. 6*H*).

### PTS modulates SIRT1-dependent ERα deacetylation to enhance transactivation activities

**Figure 7:**
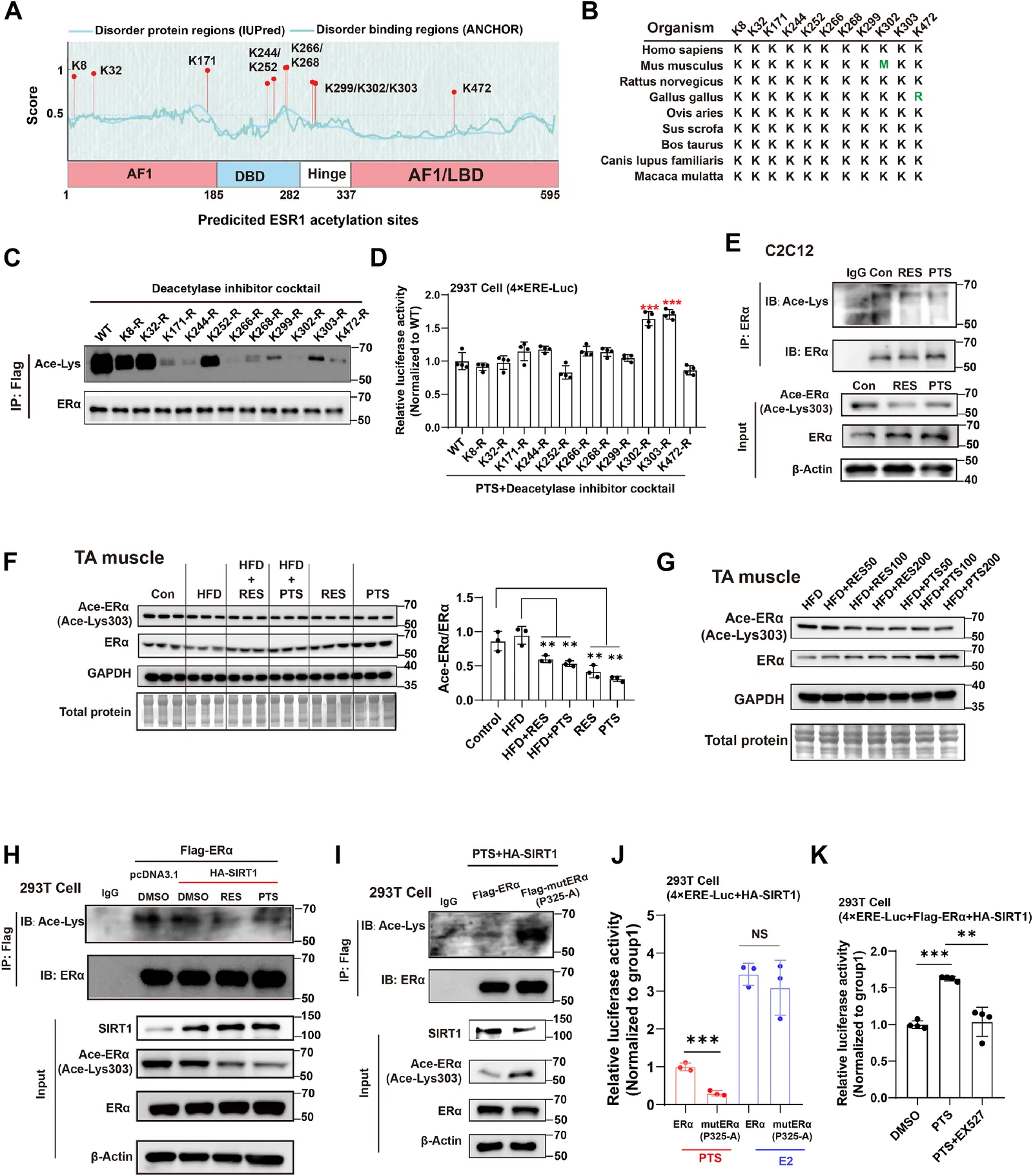
**PTS modulates SIRT1-dependent ERα deacetylation to enhance transactivation activities of ERα.***A*, predicted lysine acetylation sites of ERα were analyzed using the online tool GPS 6.0 (https://gps.biocuckoo.cn/online.php). *B*, conservation of lysine acetylation sites in ERα across different species was compared using the NCBI Multiple Sequence Alignment Viewer tool. *C*, 293T cells transfected with Flag-ERα plasmids with mutations at different lysine sites were treated with 1% deacetylase inhibitor cocktail (40 μM Trichostatin A, 1 mM EX-527, 400 mM nicotinamide, and 200 mM sodium butyrate) for 1 day. Co-IP detection of acetylated-ERα expression. *D*, 293T cells transfected with 4 × ERE-Luc and Flag-ERα plasmids with mutations at different lysine sites were treated with 2 μM PTS + 1% deacetylase inhibitor cocktail for 1 day. The cells were then collected for luciferase assays. *E*, C2C12 myotubes were treated with DMSO control, 2 μM RES or 2 μM PTS for 1 day. Co-IP detection of acetylated-ERα expression. *F* and *G*, the protein expression of ERα and acetylated-ERα (Ace-Lys303) in TA muscle. (*H*) 293T cells transfected with Flag-ERα and HA-SIRT1 (or vector) plasmids were treated with DMSO control, 2 μM RES or 2 μM PTS for 1 day. Co-IP detection of acetylated-ERα expression. *I*, 293T cells transfected with Flag-ERα (or Flag-mutERα, proline 325 to alanine substitution) and HA-SIRT1 plasmids were treated with 2 μM PTS for 1 day. Co-IP detection of acetylated-ERα expression. *J*, 293T cells transfected with 4 × ERE-Luc, Flag-ERα (or Flag-mutERα), and HA-SIRT1 plasmids were treated with 2 μM PTS or E2 for 1 day. The cells were then collected for luciferase assays. *K*, 293T cells transfected with 4 × ERE-Luc, Flag-ERα, and HA-SIRT1 were treated with DMSO control, 2 μM PTS, or 2 μM PTS + 5 μM EX527 for 1 day. Samples were collected to measure luciferase activity. For (*D*) and (*K*), n = 4 per group. For (*F*) and (*J*), n = 3 per group. One-way ANOVA followed by Duncan’s multiple range test. ∗∗*p* < 0.01, and ∗∗∗*p* < 0.001. Data are presented as means ± SD.

The acetylation status of ERα influences its transactivation activities (25, 26). As a deacetylase, SIRT1 deacetylates target proteins through protein-protein interactions. This led us to hypothesize that PTS might modulate SIRT1-dependent ERα deacetylation to enhance transactivation activities. We first predicted potential lysine acetylation sites on ERα (Fig. 7*A*). These sites were largely conserved across species, except for K302 and K472 (Fig. 7*B*). By mutating the lysine (K) to arginine (R) to mimic the deacetylated state, we identified K171, K244, K266, K268, K299, K302, K303, and K472 as potential acetylation sites on ERα (Fig. 7*C*). Luciferase reporter assays further suggested that deacetylation at K302 and K303 might enhance PTS sensitivity (Fig. 7*D*). Since K302 is naturally methionine (M) in mice, we focused on K303. In C2C12 myotubes, both RES and PTS reduced the overall acetylation status of ERα and the expression of Ace-Lys303-ERα (acetylated ERα at K303) (Fig. 7*E*). Similarly, in TA muscle, RES and PTS downregulated Ace-Lys303-ERα levels (Fig. 7, *F* and *G*).

We further investigated whether RES- and PTS-mediated ERα deacetylation depends on SIRT1. Overexpression of SIRT1 reduced both total ERα acetylation and Ace-Lys303-ERα expression, an effect that was further enhanced by RES and PTS treatment (Fig. 7*H*). Conversely, mutating the ERα-SIRT1 interaction site increased Ace-Lys303-ERα levels (Fig. 7*I*), suggesting that PTS may promote Ace-Lys303-ERα deacetylation by strengthening the ERα-SIRT1 interaction. Additionally, luciferase reporter assays showed that mutating the ERα-SIRT1 interaction site selectively impaired PTS, but not E2, ligand sensitivity (Fig. 7*J*). Inhibiting SIRT1 activity similarly diminished PTS’s ligand-sensitizing effects (Fig. 7*K*). Together, these results demonstrate that PTS specifically enhances the ERα-SIRT1 interaction, thereby promoting Ace-Lys303-ERα deacetylation and subsequently increasing transactivation activities of ERα.

## The metabolic regulatory benefits of PTS are mediated by skeletal muscle ERα/SIRT1 signaling

**Figure 8:**
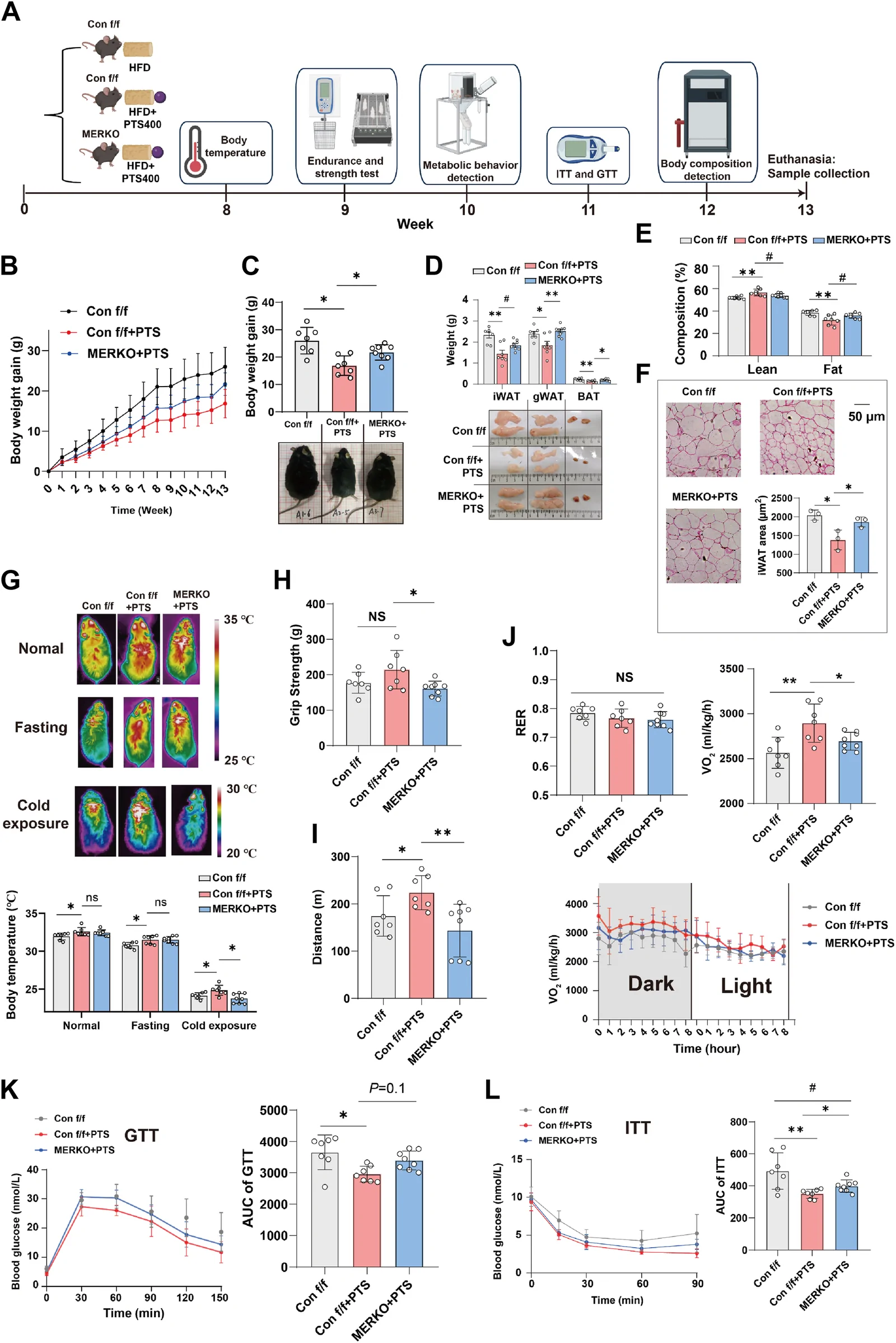
**Effects of skeletal muscle-specific ERα knockout on PTS-improved metabolic health in male mice.***A*, male mice were randomly divided into three groups: wild-type mice fed high-fat diet (HFD, n = 7) (Con f/f), wild-type mice fed HFD supplemented with 400 mg/kg PTS (Con f/f + PTS, n = 7), and skeletal muscle-specific ERα knockout mice fed HFD supplemented with 400 mg/kg PTS (MERKO + PTS, n = 8). The experiment lasted 13 weeks. Body temperature was measured at week 8, endurance and strength tests at week 9, metabolic behavior detection at week 10, insulin tolerance test (ITT) and glucose tolerance test (GTT) at week 11, and body composition analysis at week 12. *B*, body weight gain curves during the 13-weeks experimental period. *C*, body weight gain at the end of the experiment and representative photographs of mice selected based on average body weight. *D*, weights of BAT, iWAT, and gWAT, along with representative images. *E*, lean and fat composition measured by body composition analysis. *F*, H&E staining of iWAT and adipocyte area quantification. *G*, fasting and cold exposure body surface temperature. *H*, grip strength measured by grip strength test. *I*, running distance in endurance test. *J*, respiratory exchange ratio (RER), average hourly oxygen consumption (VO2), and VO2 over time curves. *K* and *L*, blood glucose levels at different time points and corresponding area under the curve (AUC) in GTT (*K*) and ITT (*L*). For (*F*), n = 3 per group. For all other panels, n = 7 per group in Con f/f group and Con f/f + PTS group and n = 8 per group in MERKO + PTS group. One-way ANOVA followed by Duncan’s multiple range test. ^#^*p* < 0.1, ∗*p* < 0.05, and ∗∗*p* < 0.01. Data are presented as means ± SD.

We constructed male mice with skeletal muscle-specific ERα knockout to comprehensively investigate whether ERα mediates the metabolic regulatory benefits of PTS in HFD-fed mice (Fig. 8*A*). Consistent with prior observations, PTS treatment did not alter *Esr1* gene expression but enhanced *Sirt1* gene expression (Fig. S12, *A* and *B*). In contrast, ERα knockout reduced gene and protein and expression of both ERα and SIRT1 in skeletal muscle compared to WT mice (Fig. S12, *A* and *B*), which validates our knockout mode. PTS supplementation reduced body weight (Fig. 8, *B* and *C*), fat tissue weight (Fig. 8*D*), decreased fat composition and promoted lean composition (Fig. 8*E*), and reduced adipocyte area in iWAT (Fig. 8*F*). However, skeletal muscle-specific ERα knockout attenuated these effects of PTS (Fig. 8, *B*–*F*). PTS promoted basal, fasting, and cold-exposed body temperature; while skeletal muscle-specific ERα knockout abolished the promotion of PTS on cold-exposed body temperature (Fig. 8*G*). Additionally, PTS supplementation had no effect on grip strength but improved endurance performance, whereas ERα knockout reduced endurance performance and grip strength compared to the PTS-supplemented group (Fig. 8, *H* and *I*). Furthermore, PTS supplementation increased oxygen consumption and lowered blood glucose levels in GTT and ITT, and skeletal muscle-specific ERα knockout similarly attenuated these effects of PTS (Fig. 8, *J–L*). Regarding the skeletal muscle oxidative phenotype, ERα knockout attenuated the PTS-induced conversion of fast to slow twitch muscle fibers (Fig. S12, *C–E*) and diminished its promotive effects on mitochondrial complex protein expression (Fig. S12*F*).

To assess whether the metabolic regulatory benefits of PTS also extend to females, we also generated female mice with skeletal muscle-specific ERα knockout. In HFD-fed female mice, PTS treatment and ERα knockout had no significant effects on body weight (Fig. S13, *A* and *B*), fat tissue weight (Fig. S13*C*), or body composition (Fig. S13*D*). Regarding thermogenesis, PTS promoted cold-exposed body temperature in female mice, but ERα knockout did not attenuate this effect of PTS (Fig. S13*E*). Interestingly, PTS improved grip strength in female mice, whereas ERα knockout abolished this effect (Fig. S13*F*). In addition, PTS had no significant effect on endurance performance in female mice, while ERα knockout reduced endurance performance (Fig. S13*G*). Furthermore, neither PTS nor ERα knockout significantly affected respiratory exchange ratio, oxygen consumption, ITT, or GTT in HFD-fed female mice (Fig. S13, *H–J*).

Moreover, in C2C12 myotubes, inhibition of either ERα or SIRT1 blocked the PTS-induced upregulation of ERα, SIRT1, and p-AMPK expression (Fig. S14*A*), abolished the PTS-promoted shift from fast to slow muscle fibers (Fig. S14, *B–D*), and impeded the PTS-induced increase in mitochondrial number (Fig. S14*E*) as well as mitochondrial complex protein expression (Fig. S14*F*). Additionally, siRNA-mediated knockdown of ERα abolished the PTS-induced upregulation of SIRT1 expression and the promotion of fast-to-slow muscle fiber type transition (Fig. S14*G*). In summary, the section demonstrates that skeletal muscle ERα/SIRT1 signaling mediates the metabolic regulatory benefits of PTS under HFD.

## Discussion

As a dimethyl ether analog of RES, PTS exhibits superior metabolic stability and lipophilicity due to its methoxy substitutions compared to RES, thereby enhancing its bioavailability and pharmacological activity (11). While both RES and PTS have been individually demonstrated to improve metabolic disorders (13), comparative studies evaluating their efficacy as metabolic regulators remain limited. In this study, we comprehensively compared the functional effects of dietary RES and PTS as metabolic regulators, including promoting skeletal muscle oxidative phenotype and mitochondrial function, inducing thermogenesis, enhancing endurance, resisting obesity, and improving glucose homeostasis. Unexpectedly, under our experimental conditions, it was found that at a dose of 400 mg/kg diet, RES and PTS exhibited comparable metabolic regulatory effects. We speculate that at high doses, both RES and PTS may reach saturating pharmacological concentrations, masking their differences. To further compare their dose-dependent effects, we reduced the supplementation levels and found that PTS indeed acts as a more potent metabolic regulator than RES at lower doses. Specifically, RES required at least 100 mg/kg diet (approximately equivalent to 12 mg/kg BW/day) to elicit metabolic regulatory effects, whereas PTS at 50 mg/kg diet (approximately equivalent to 6 mg/kg BW/day) was sufficient to produce similar benefits. Consistent with our findings, a previous study reported that supplementation with 30 mg/kg BW/day of either RES or PTS (roughly corresponding to 200 mg/kg diet in our study) exerted comparable effects in counteracting obesity and preventing fatty liver (27). Notably, at the lower dose of 15 mg/kg BW/day, PTS demonstrated hepatoprotective effects against fatty liver, whereas RES showed no such efficacy (27, 28). The pharmacological saturation observed at higher doses for RES or PTS may result from relative saturation of phase II metabolizing enzymes (11) or achievement of maximal SIRT1 activation capacity.

As plant-derived compounds, both RES and PTS exhibit low toxicity. Toxicological studies have demonstrated that long-term supplementation with 500 mg/day RES (29) or 250 mg/day PTS (30) is safe for humans. According to dose translation between mice and humans (31), a dietary 100 mg/kg RES or PTS is approximately equivalent to a daily intake of 70 mg in adults, which is below the safety thresholds. Given that metabolic diseases are chronic conditions requiring long-term intervention, and considering human dietary habits, it is recommended to incorporate RES or PTS as food additives or to consume foods naturally rich in these compounds (such as grapes and blueberries) for the management of metabolic disorders. Notably, while current clinical applications of RES for metabolic disorders and obesity show limited efficacy, our findings suggest PTS may serve as a potential alternative. However, clinical research on PTS for metabolic disorders remains limited and requires further investigation.

Integrating target prediction and PPI analysis, we preliminarily hypothesize that ERα may serve as an intermediate signaling factor through which RES and PTS activate SIRT1. Estrogens exert their effects *via* estrogen receptors, with the classical estrogen receptors including ERα and ERβ. Indeed, it has been well-documented that RES and its analogs act as phytoestrogens to activate ERα (32). In MCF-7 cells with high ERα expression, RES induces stronger ERα activation than E2 (33). Furthermore, RES analogs have demonstrated ERα-activating activity (34). Structural analyses reveal that RES binds to ERα, thereby altering the coactivator-binding surface of AF2 in ERα and recruiting coactivators to activate ERα (35). In our study, the ability of RES and PTS to bind and activate ERα was validated through MD simulations, SPR, and luciferase reporter assays. RES and the synthetic estrogen diethylstilbestrol share a stilbene backbone, which may serve as the structural basis for the estrogenic effects of RES (32). Similarly, in our luciferase reporter assays, it was observed that only plant-derived SIRT1 activators containing a stilbene backbone—such as RES, PTS, cis-RES, and butein—activated ERα, whereas synthetic SIRT1 activators like E1231 and SIRT1 activator three did not. It is worth noting that RES and PTS showed comparable Kd values for ERα binding in the SPR assay, implying that the enhanced potency of PTS is more likely attributable to its superior metabolic stability and increased ability to cross cell membranes.

Current evidence suggests that SIRT1 functions as a downstream target of ERα in mediating estrogen signaling. It is observed that ERα-positive cells express higher SIRT1 levels than ERα-negative cells (36). Furthermore, E2 treatment enhances SIRT1 expression in an ERα-dependent manner in both ZR75.1 breast cancer cells and 3T3-L1 adipocytes (36, 37), while adipose tissue-specific ERα knockout leads to reduced SIRT1 expression (37). However, the precise molecular mechanisms underlying ERα-mediated regulation of SIRT1 expression remain incompletely understood, particularly with respect to the potential roles of RES and PTS in this process. Previous studies have demonstrated that in cells transfected with ERα, estradiol (E2) treatment enhances SIRT1 promoter-specific fluorescent protein fluorescence and luciferase activity (36). In our study, RES and PTS promoted the expression of the *Sirt1* gene but had no effect on the *Esr1* gene expression, which suggests that PTS may enhance *Sirt1* transcription *via* ERα. The hypothesis was subsequently confirmed by our EMSA and dual-luciferase assays in our study. However, we cannot exclude the possibility that RES and PTS may also regulate SIRT1 expression through other transcriptional factors. For example, previous studies have reported that RES and PTS exert their beneficial effects partly through activation of Nrf2 signaling (38, 39), and Nrf2, as a transcription factor, has been implicated in promoting SIRT1 transcription (40).

In addition, PPI serves as a crucial mechanism regulating protein stability and expression. PPI analysis suggests a potential interaction between ERα and SIRT1, while RES and PTS promote their expression in a significantly positive correlation. Based on these findings, it is hypothesized that the interaction between ERα and SIRT1 promotes protein stability, thereby enhancing their expression. The ERα-SIRT1 interaction has been previously demonstrated in MCF7 and ZR75.1 cells, where Co-IP and IF confirmed their physical association and nuclear co-localization, with E2 further enhancing this interaction (36). Additionally, a recent study identified 1655 potential SIRT1-interacting proteins *via* IP-MS, with Co-IP validation confirming ERα among 157 proteins that directly bind SIRT1 (41). In our study, we identified RES and PTS as stabilizers of the ERα-SIRT1 interaction, which increased the protein stability of ERα and SIRT1. Intriguingly, MD simulations revealed that natural SIRT1 activators with a stilbene structure exhibited superior compatibility as ERα-SIRT1 stabilizers compared to synthetic SIRT1 activators, though further experimental validation is required. Moreover, the MD simulations-based workflow established here provides a scalable platform for high-throughput virtual screening of ERα-SIRT1 interaction stabilizers, which will be the focus of our future investigations.

Studies have demonstrated that interactions between estrogen receptors and certain proteins (*e*.*g*., MUC1, PIN1, GSK3, LMTK3, and ABL) can mask ubiquitination sites on estrogen receptors, thereby preventing its ubiquitin-mediated degradation and leading to increased estrogen receptors protein levels (42). Similarly, we speculate the enhanced ERα-SIRT1 interaction may promote their stability by impeding ubiquitination. During ubiquitination, the E1 activating enzyme transfers ubiquitin to the E2 conjugating enzyme, after which the ubiquitin ligase E3 recognizes the target protein and facilitates the transfer of ubiquitin from E2 to the substrate (43). Our predictions identified MDM2 as a shared E3 ligase for both ERα and SIRT1, which has been separately demonstrated in previous studies (44, 45). Notably, MD simulations revealed that MDM2 binds to the interaction interfaces of ERα and SIRT1, suggesting their mutual binding may block MDM2-mediated ubiquitination and consequently enhance protein stability, a mechanism we experimentally validated in this study.

The acetylation status of different lysine sites on ERα may affect the transactivation activities of the ERα. It has been reported that acetylation of K266 and K268 enhances transactivation activities (25), while acetylation of K302 and K303 reduces transactivation activities (26). In our study, PTS promotes the deacetylation of K303 on ERα by enhancing the interaction between ERα and SIRT1, which may contribute to the PTS-dependent transactivation activities. However, increased acetylation of ERα may enhance its protein stability by inhibiting the ubiquitination process, which could involve competitive modification between acetylation and ubiquitination at the same lysine residues (46). Studies have shown that acetylation of ERα at sites such as K266, K268, and K299 suppresses its ubiquitination, thereby promoting ERα stability (47). This suggests that the deacetylation process mediated by SIRT1 might reduce ERα protein stability, which contradicts the previous finding of enhanced protein stability. However, in our view, blocking E3 ligase recognition likely exerts a more profound effect on ubiquitination than site-specific deacetylation, as it directly disrupts the entire ubiquitination cascade rather than merely modulating individual lysine residues. A similar phenomenon has been reported for SIRT6, which deacetylates ERα at K171 and K299 but paradoxically increases ERα protein stability (48).

Estrogen receptors play an important role in regulating obesity, insulin sensitivity, and diabetes (49). In male mice, skeletal muscle-specific knockout of ERα leads to increased fat mass (50), impaired muscle contractile performance (51), and insulin resistance (52). In our study, although knockout of skeletal muscle ERα completely blocked the effects of PTS on endurance and skeletal muscle oxidative expression, it only partially weakened the effects of PTS on obesity and insulin resistance. This suggests that the beneficial effects of PTS on metabolic health may be partially mediated by ERα signaling in adipose tissue (53). However, adipose-specific ERα knockout was not performed in the current study due to our primary focus on skeletal muscle ERα, which may represent a limitation of this study. The ERα/SIRT1 axis mediates diverse physiological processes, including autophagy regulation and obesity (37), cardiac hypertrophy (54), breast cancer (55), ischemic brain injury (56), and acute kidney injury (57). Here, we provide evidence that PTS acts as a metabolic regulator through skeletal muscle ERα/SIRT1 signaling, as demonstrated by the complete blockade of PTS-induced SIRT1 upregulation upon ERα knockout and the abolition of PTS-mediated fast-to-slow muscle fiber-type transition following inhibition of ERα/SIRT1 signaling.

For female mice, neither PTS treatment nor skeletal muscle-specific ERα knockout affected obesity or metabolic phenotypes. The sex dimorphism in metabolic disorders is widely reported, with male mice more susceptible than females, a difference partly attributed to estrogen. Female mice have high endogenous estrogen levels that saturate and activate estrogen receptors, conferring resistance to obesity and improving metabolic health (58). Consequently, in females, exogenous PTS, acting as an ERα agonist, may have difficulty producing detectable additive effects beyond the already high baseline activity. Furthermore, in female mice, skeletal muscle-specific ERα knockout only impairs endurance and strength performance, functions closely linked to skeletal muscle, without significantly affecting energy metabolism or obesity. This finding aligns with previous reports, where ERα deficiency in skeletal muscle does not alter body composition, energy expenditure, or mitochondrial function under HFD (59). In contrast, overexpression of ERα in skeletal muscle enhances endurance (60), whereas skeletal muscle-specific ERα knockout impairs the oxidative phenotype and mitochondrial function of skeletal muscle (61). Collectively, these sex-specific results further support the conclusion that PTS exerts its metabolic regulatory effects through the ERα signaling pathway in male mice.

In summary, our study found that PTS is a more potent metabolic regulator than RES in promoting skeletal muscle oxidative phenotype and counteracting obesity in male mice. More significantly, our study demonstrates that the upregulation of SIRT1 expression induced by RES and PTS may be partially mediated by ERα. These findings highlight the therapeutic potential of PTS as a superior substitute for RES in metabolic disorders. Additionally, targeted stabilization of the ERα-SIRT1 interaction may represent a promising therapeutic strategy for developing novel metabolic regulators for metabolic health enhancement.

## Experimental procedures

### Animal model and experiment

All procedures of animal experiments were performed according to protocols approved the Animal Care Advisory Committee of Sichuan Agricultural University under permit No. YYS20230727.

Wild-type C57BL/6J mice were purchased from Beijing Vital River Laboratory Animal Technology. *Esr1*-floxed mice were crossed with a muscle-specific Cre transgenic mouse (MCK-Cre) to generate skeletal muscle-specific ERα knockout mice (MCK-Cre-ERα f/f) and negative control mice (Con f/f). These gene-edited mice were provided by Shanghai Model Organisms Center and were further bred in our laboratory. All mice were housed individually (23 °C ± 2 deg, 12-h light/12-h dark cycle) and had free access to food and water. Body weight and food intake were measured weekly.

For comparison of single-dose RES and PTS effects under both standard chow and high-fat diet (HFD) conditions, male mice were randomly allocated into six groups (n = 20 per group): chow diet control, HFD control, HFD supplemented with 400 mg/kg RES, HFD supplemented with 400 mg/kg PTS, chow diet supplemented with 400 mg/kg RES, and chow diet supplemented with 400 mg/kg PTS.

For dose-response evaluation of RES and PTS under HFD, male mice were divided into seven groups (n = 8-9 per group): HFD control, HFD supplemented with 50, 100, or 200 mg/kg RES, and HFD supplemented with 50, 100, or 200 mg/kg PTS.

To investigate the effects of skeletal muscle-specific ERα knockout, male and female mice were separately subjected to the same experimental design. Within each sex, mice were randomly divided into three groups: wild-type mice fed HFD (n = 7), wild-type mice fed HFD supplemented with 400 mg/kg PTS (n = 7), and skeletal muscle-specific ERα knockout mice fed HFD supplemented with 400 mg/kg PTS (n = 8).

All experiment lasted 13 weeks. At the end of the experiment, mice were anesthetized with an intraperitoneal injection of Zoletil (25 mg/kg body weight; Zoletil 50; Virbac, Nice, France), followed by cardiac blood collection and euthanasia. The following adipose and skeletal muscle tissues were collected, weighed, and processed for further analysis: tibialis anterior (TA), gastrocnemius (GAS), soleus (SOL), brown adipose tissue (BAT), inguinal white adipose tissue (iWAT), and gonadal white adipose tissue (gWAT). After photographic documentation, all tissue samples were immediately snap-frozen and stored at −80 °C for subsequent experiments.

### Cell culture

C2C12 cells (Shanghai Cell Bank) were cultured in Dulbecco's Modified Eagle medium (DMEM) (Invitrogen) supplemented with 10% fetal bovine serum (FBS, GibcoK) and 1% penicillin-streptomycin (P-S, Gibco). When cells reached 80% confluence, the medium was replaced with fresh medium containing 2% horse serum (Gibco) (replacing the original 10% FBS) to induce differentiation.

3T3-L1 cells (Pricella) were cultured in DMEM supplemented with 10% FBS and 1% P-S. When the cells reached 70% confluence, the medium was replaced with MDI induction medium (basal DMEM supplemented with 0.5 mM IBMX (Sigma), 1 μM dexamethasone (Sigma), and 10 μg/ml insulin (Sigma)) to induce cell differentiation for 3 days. Subsequently, the medium was replaced with insulin medium (basal DMEM supplemented with 10 μg/ml insulin) to further induce differentiation for an additional 3 days. After a total of 6 days of differentiation, the medium was changed back to basal DMEM for continued culture.

HEK-293T cells (Pricella) were maintained in DMEM supplemented with 10% FBS and 1% P-S. Upon reaching 80% confluence, the cells were transfected into the plasmid using Lipo8000 (C0533FT, Beyotime) transfection reagent according to the manufacturer’s protocol. Following transfection, the culture medium was replaced with Opti-MEM reduced-serum medium for continued incubation.

### Genotype identification

Genomic DNA was extracted from mouse tail tips using a standard phenol-free protocol. Briefly, tail samples were lysed overnight at 56 °C in 0.5 ml of lysis buffer containing 50 μl proteinase K. After centrifugation (12,000 rpm, 10 min, room temperature), the supernatant was transferred to a new tube, mixed with 1 ml ethanol to precipitate DNA, and centrifuged again (12,000 rpm, 15 min, 4 °C). The pellet was washed with 75% ethanol (12,000 rpm, 5 min, 4 °C), air-dried briefly, and resuspended in nuclease-free water. The forward primer (5′-TGGTAATGCCAGGGAAGCTG-3′) and reverse primer (5′-CAGGAGAATGAGGTGGCACA-3′) amplify the Flox region of the *Esr1* gene for *Esr1*-Flox genotyping (wild type: 396 bp; heterozygote: 396 bp and 447 bp; homozygote: 447 bp). For MCK-Cre transgenic mouse genotyping, the forward primer (5′-GTGAAACAGCATTGCTGTCACTT-3′) and reverse primer (5′-TAAGTCTGAACCCGGTCTGC-3′) target the Cre recombinase gene (transgenic band: 450 bp), while the internal positive control primers (Forward: 5′-CAAATGTTGCTTGTCTGGTG-3′, Reverse: 5′-GTCAGTCGAGTGCACAGTTT-3′) produce a 200 bp control band. The PCR reaction mixture with a total volume of 20 μl contains nuclease-free water, 2 × Taq Plus Master Mix (P112-01, Vazyme, Nanjing, China), primers, and genomic DNA at 50 to 100 ng/μl concentration. After thorough mixing, PCR amplification is performed using the following program (1): 94 °C for 3 min (2), 94 °C for 30 s (3), 60 °C for 30 s (4), 72 °C for 30 s (34 cycles of steps 2–4) (5), 72 °C for 5 min (6), Hold at 12 °C.

### Glucose tolerance test (GTT) and insulin tolerance test (ITT)

GTT was conducted after an overnight fasting period (8:00 PM–8:00 AM). Glucose powder was dissolved in sterile saline to prepare a 40% (w/v) solution, which was administered *via* intraperitoneal injection at a dose of 1.5 g/kg body weight. Blood glucose levels of tail vein blood samples were measured using a glucometer (GA-3, Sinocare Inc) at 30, 60, 90, and 120 min post-injection. ITT was performed after a 4-h fasting period (6:00 AM–10:00 AM). Insulin was administered *via* intraperitoneal injection at a dose of 1 U/kg body weight. Blood glucose levels of tail vein blood samples were measured using the same glucometer at 15, 30, 60, and 90 min post-injection.

### Endurance running capacity test

The endurance running capacity of mice was tested using a treadmill (XR-PT-10B, Xinruan, Shanghai, China). Mice were acclimated by running at 5 m/min for 5 min daily on a flat surface for three consecutive days. For the test, the treadmill was set to a 5° incline. Mice started running at 5 m/min for 10 min, with the speed increased by 2 m/min every 5 min thereafter. Exhaustion was defined as the inability to continue running despite 0.3 mA electrical stimulation. The time to exhaustion and total running distance and time were recorded for data analysis.

### Grip strength test

The grip strength of mice was measured using a Grip Strength Meter (XR501, Xinruan, Shanghai, China). During testing, each mouse was held with its torso parallel to the metal grid of the device. A uniform pulling force was gently applied through the tail until the mouse was pulled away from the grid. The maximum grip strength was recorded. This measurement was repeated three times for each animal, with the highest value among the three trials being recorded as the final grip strength.

### Rectal and surface temperature measurement under cold exposure and fasting

For cold exposure, mice were housed individually in a temperature-controlled chamber set at 4 °C. Temperatures were measured prior to cold exposure (at room temperature 22 °C ± 2 deg) and at regular intervals (1, 2, and 3 h) during the cold exposure period. For fasting, mice were deprived of food overnight (8:00 PM–8:00 AM) but allowed free access to water, temperatures were measured before and after fasting under standard room temperature (22 °C ± 2 deg). Rectal temperature was measured using a digital rectal thermometer (RET-3 rectal probe), while surface temperature was recorded simultaneously using a handheld infrared thermal imaging camera (H21, HIKIMICRO). Surface temperature data were analyzed and pseudo-color images were generated using the HIKIMICRO analyzer.

### Energy metabolism measurements

The energy metabolism monitoring system (EM-8M-WAG, TOW-INT Tech.) was used to measure oxygen consumption (VO_2_) and the respiratory exchange ratio (RER). The system consists of eight independent metabolic chambers, each housing a single mouse. Prior to formal measurements, mice were acclimated to the chambers for 3 days. Subsequently, VO_2_ and RER were monitored under 12-h light/12-h dark cycles. To exclude potential instability during system initialization and termination, data from the middle 8 h of both light and dark phases were selected for analysis and presentation.

### Seahorse XF analysis

Differentiated C2C12 myotubes were treated with DMSO control, 2 μM RES, or 2 μM PTS for 1 day, and the same treatment conditions were applied to all panels. Oxygen consumption rate (OCR) and extracellular acidification rate (ECAR) were measured using the Seahorse XF96 extracellular flux analyzer (Agilent Technologies) with the XF Cell Mito Stress Test Kit (Cat. No. 103015-100) and XF Cell Glycolysis Stress Test Kit (Cat. No. 103020-100), respectively. OCR was assessed by sequential injection of oligomycin, FCCP, and rotenone/antimycin A (ROT/AA), whereas ECAR was measured following sequential injection of glucose, oligomycin, and 2-deoxyglucose (2-DG). The final concentrations were 1.5 μM oligomycin, 1.5 μM FCCP, and 0.5 μM ROT/AA for OCR analysis, and 10 mM glucose, 2 μM oligomycin, and 50 mM 2-DG for ECAR analysis.

### Body composition analysis

Body composition (lean and fat) of mice was measured using a quantitative magnetic resonance system (QMR06-090H-PRO, Niumag Corporation). Prior to measurement, each mouse was weighed for weight calibration. Mice were gently restrained in cylindrical holders and inserted into the imaging chamber for analysis.

### Protein extraction and Western blot

For tissue total protein extraction, 1 ml of RIPA Lysis Buffer (P0013B, Beyotime) was added to 100 mg of tissue, followed by the addition of steel beads. Homogenization was performed using a magnetic homogenizer, and the supernatant was collected by centrifugation at 12,000 rpm for 10 min. For total cell protein extraction, RIPA Lysis Buffer was added to cells plates after removing the culture medium, and the supernatant was collected by centrifugation at 12,000 rpm for 10 min. For nuclear protein extraction, NE-PER Nuclear and Cytoplasmic Extraction Reagents (78,833, Thermo Fisher) were used according to the manufacturer's instructions. The protein concentration of the extracted supernatants was determined using a BCA Protein Assay Kit (P0012, Beyotime), and all samples were diluted to the same concentration with RIPA Lysis Buffer. SDS-PAGE Sample Loading Buffer (P0015, Beyotime) was then added to the samples, which were heated at 98 °C for 10 min in a metal bath to complete sample preparation. The prepared samples were subjected to electrophoresis on a Bis-Tris gel, followed by protein transfer to a PVDF membrane using a wet transfer system. The PVDF membranes were blocked with 5% non-fat milk for 1.5 h at room temperature, incubated with primary antibodies overnight at 4 °C, and then incubated with the corresponding secondary antibodies for 1 h at room temperature.

The primary antibodies used included: rabbit polyclonal anti-PPARγ (Cell Signaling Technology (CST), Cat# 2430), rabbit polyclonal anti-HSL (CST, Cat# 4107), rabbit polyclonal anti-p-HSL (CST, Cat# 4139), rabbit polyclonal anti-NDUFA9 (Mitochondrial complex I, GeneTex, Cat# GTX132978), rabbit monoclonal anti-SDHA (Mitochondrial complex II, GeneTex, Cat# GTX636098), mouse monoclonal anti-UQCRC1 (Mitochondrial complex III, GeneTex, Cat# GTX630393), rabbit monoclonal anti-MTCO1 (Mitochondrial complex IV, Bioss, Cat# bs-3953R), rabbit polyclonal anti-ATP5B (Mitochondrial complex V, GeneTex, Cat# GTX132925), rabbit polyclonal anti-Slow MyHC (ServiceBio, Cat# GB111857), rabbit polyclonal anti-Fast MyHC (ServiceBio, Cat# GB112130), rabbit polyclonal anti-FASN (CST, Cat# 3189), rabbit polyclonal anti-UCP1 (Proteintech, Cat# 23673-1-AP), mouse monoclonal anti-ERα (Santa Cruz, Cat# sc-8002), rabbit polyclonal anti-ERα (Acetyl Lys303) (UPT Biotechnology, Cat# PLN000109), rabbit polyclonal anti-SIRT1 (Proteintech, Cat# 13161-1-AP), rabbit monoclonal anti-AMPK (CST, Cat# 5831), rabbit monoclonal anti-p-AMPK (CST, Cat# 2535), rabbit polyclonal anti-acetylated-lysine (CST, Cat# 9441), mouse monoclonal anti-Histone H3 (Beyotime, Cat# AF0009), mouse monoclonal anti-Ub (CST, Cat# 3936), mouse monoclonal anti-HA (Beyotime, Cat# AH158), rabbit monoclonal anti-Flag (CST, Cat# 14793), rabbit polyclonal anti-MDM2 (OmnimAbs, Cat# OM284455), rabbit polyclonal anti-GAPDH (ServiceBio, Cat# GB11002), and mouse polyclonal anti-β-actin (ServiceBio, Cat# GB12001). The secondary antibodies used included HRP-labeled goat anti-rabbit IgG(H + L) (Beyotime, Cat# A0208) and HRP-labeled goat anti-mouse IgG (H + L) (Beyotime, Cat# A0216). All primary antibodies were diluted at 1:1000, and secondary antibodies at 1:3000. HRP signals were detected using the BeyoECL Moon kit (P0018FS, Beyotime), and signals were captured using a ChemiDoc Imaging System (Bio-Rad). Band densities were quantified using Image Lab 5.1 software (Bio-Rad). Coomassie brilliant blue staining of total protein and GAPDH were used as dual internal controls for tissue samples, while β-actin was used as the internal control for cell samples.

### Co-immunoprecipitation (Co-IP)

Cell samples were lysed using Cell Lysis Buffer for Western and IP (P0013, Beyotime). Anti-Flag (P2115, Beyotime) or anti-HA magnetic beads (P2121, Beyotime) were washed with TBS buffer and resuspended for direct use. For Protein A + G magnetic beads (P2108, Beyotime), antibodies or IgG were added and incubated with the beads at room temperature for 30 min to allow binding. Protein lysates were incubated overnight at 4 °C with these magnetic beads. After incubation, magnetic beads were separated using a magnetic rack, washed thoroughly with TBS, and resuspended in SDS-PAGE Sample Loading Buffer (P0015, Beyotime). The samples were heated at 95 °C for 5 min to elute bound proteins. Beads were magnetically separated, and the supernatant was collected.

### Gene expression and mtDNA copy number *via* quantitative PCR (qPCR)

RNA was extracted using the RNA Isolater Total RNA Extraction Reagent (R401-01, Vazyme) following the manufacturer’s instructions. The extracted RNA was reverse-transcribed into cDNA using the HiScript II Q RT SuperMix for qPCR (R223-01, Vazyme). Genomic DNA was isolated using the Mammalian Genomic DNA Extraction Kit (D0061, Beyotime) for mtDNA copy number determination. qPCR was performed using ChamQ SYBR Color qPCR Master Mix (Q411-02, Vazyme) on a 7900 HT Real-Time PCR System (Applied Biosystems) with a 10 μl reaction volume loaded onto a 384-well block. For mtDNA copy number quantification, relative mtDNA levels were measured by qPCR using primers specific to the mitochondrially encoded *ND1* gene and normalized to the nuclear-encoded *Rplp0* gene. For tissue expression profiling of the *Esr1* gene, *Arf1* was selected as the internal reference. Primer sequences are provided in Table S3.

### Paraffin section preparation and HE staining of adipose tissue

Adipose tissue samples were immersed in a specialized fixative for adipose tissues, with the samples pressed into the fixative using absorbent cotton to ensure thorough fixation. Tissues were fixed for over 24 h at room temperature, trimmed, and placed into embedding cassettes. Dehydration was performed through a graded alcohol series (75%, 85%, 90%, and 95% ethanol, followed by absolute ethanol, ethanol-xylene, and xylene), and the samples were infiltrated with molten paraffin at 65 °C. The tissues were embedded, cooled on a −20 °C freezing platform, and trimmed into paraffin blocks. Sections were cut at a thickness of 4 μm, floated on a 40 °C water bath, mounted onto slides, and dried at 60 °C. For HE staining, sections were deparaffinized in xylene, rehydrated through ethanol gradients, and stained with hematoxylin for 3 to 5 min. After differentiation in acid alcohol and bluing in ammonia water, sections were counterstained with eosin for 5 min. Finally, the sections were dehydrated in absolute ethanol, cleared in xylene, and mounted with neutral resin for microscopic examination.

### Transmission electron microscopy (TEM)

Fresh muscle tissues (1 mm^3^) were fixed in TEM fixative, post-fixed with 1% osmium tetroxide, and dehydrated through an ethanol-acetone series (30%, 50%, 70%, 80%, 95%, and 100%) for 20 min each. Samples were infiltrated with epoxy resin 812 at 37 °C overnight, polymerized at 60 °C for 48 h, and ultrathin-sectioned (60–80 nm) using an ultramicrotome. Sections were double-stained with 2% uranyl acetate and lead citrate prior to TEM examination.

### Immunofluorescence (IF) staining

Cells were washed with PBS and fixed with Immunol Staining Fix Solution (P0098, Beyotime) for 20 min, followed by permeabilization with 0.5% Triton X-100 (P0096, Beyotime) for 20 min. Blocking was performed with QuickBlock Blocking Buffer (P0260, Beyotime) for 2 h at 37 °C. Cells were then incubated with primary antibodies overnight at 4 °C, followed by incubation with fluorescent secondary antibodies for 2 h at 37 °C. For dual immunofluorescence labeling of ERα and SIRT1, cells were incubated with a mixture of mouse monoclonal anti-ERα (1:25, Santa Cruz, Cat# sc-8002) and rabbit polyclonal anti-SIRT1 (1:50, Proteintech, Cat# 13161-1-AP) as primary antibodies, followed by secondary antibodies: Cy3-goat anti-rabbit IgG (1:200, A0521, Beyotime) and FITC-goat anti-mouse IgG (1:200, A0568, Beyotime). For Slow MyHC detection, cells were incubated with rabbit polyclonal anti-Slow MyHC (1:50, ServiceBio, Cat# GB111857) as the primary antibody and FITC-labeled Goat Anti-Rabbit IgG (1:200, Beyotime, Cat# A0562) as the secondary antibody. Finally, cell nuclei were stained with 4,6-diamidino-2-phenylindole (C1002, Beyotime) for 10 min at room temperature. Fluorescence signals were visualized and captured using a Leica DMI4000 B fluorescence microscope.

### Mitochondrial staining in live cells

MitoTracker Red CMXRos (C1049B, Beyotime) was used to specifically label bioactive mitochondria in live cells. Cell culture medium was removed and replaced with PBS containing 200 nM MitoTracker Red CMXRos. Cells were incubated in the cell culture incubator for 20 min. Fluorescence signals were captured using a Leica DMI4000B fluorescence microscope.

### Construction of the SIRT1 activator library, target prediction, and protein–protein interaction (PPI) analysis

A SIRT1 activator library was constructed by integrating commercially available SIRT1 activators from Targetmol, MedChemExpress, and Selleck (Table S1). The structures of all small molecules were downloaded in sdf format from PubChem (https://pubchem.ncbi.nlm.nih.gov/) and submitted to the PharmMapper online platform (http://lilab-ecust.cn/pharmmapper/index.html) (62) for drug target prediction using a pharmacophore-based mapping approach. The predicted candidate targets were further analyzed using the STRING database (https://cn.string-db.org/) for PPI analysis with the confidence threshold set to the highest confidence score of 0.9.

### Molecular docking and molecular dynamics (MD) simulation

The Protein Data Bank (PDB, https://www.rcsb.org/) was used to retrieve the protein structures of ERα (PDB ID: 1X7R) and SIRT1 (PDB ID: 4ZZJ). For small molecule-protein docking and MD simulations, the docking structures were optimized, and binding pockets were defined. Molecular docking was performed using AutoDock and visualized using Discovery Studio 2019 (DS2019, Dassault Systèmes, San Diego, USA). MD simulations were conducted using GROMACS (version 2022.6-GPU) (63). The conformation with the highest binding affinity from molecular docking served as the initial structure for the MD simulation. The topology file for small molecule were generated using the AMBER GAFF force field *via* the ACPYPE tool (64), while the topology file for the protein was prepared using the AMBER 99SB-ILDN force field. The system was prepared by defining the simulation box, solvating with water, neutralizing with counter ions, and performing energy minimization. Equilibration was carried out in NVT and NPT ensembles, followed by a 50 ns MD simulation under constant temperature and pressure. The final 5 ns trajectory was used to calculate MMPBSA binding energy with gmx_mmpbsa. Conformational stability was verified by root mean square deviation (RMSD) analyses. Binding modes and interactions were visualized using VMD (65).

The prediction of ERα-SIRT1 interaction stabilizers was conducted following a previously reported protocol with modifications (24). The process was divided into the following steps: First, ZDOCK was used to predict the binding conformations of ERα and SIRT1 and the top-scoring conformation was selected for further analysis. Second, the interface pocket of the selected conformation was defined using DS2019. Third, molecular docking was performed and binding free energies were calculated using DS2019. Optimal conformations were selected based on (1): high-affinity binding to the ERα/SIRT1 interface pocket, and (2) dual-binding capability (similar binding energies for ERα and SIRT1). Fourth, the best conformation in molecular docking served as the initial structure for 50 ns MD simulation in GROMACS, followed by MMPBSA binding energy calculations using gmx_mmpbsa.

### Surface plasmon resonance (SPR) detection

Chip activation was performed by injecting a freshly prepared mixture of 400 mM EDC and 100 mM NHS into Fc1 and Fc4 channels for 800 s at a flow rate of 10 μl/min. Recombinant human ERα (20 μg/ml in 10 mM NaAc, pH 5.0) was immobilized on Fc4 for 1500 s at the same flow rate, achieving an immobilization level of approximately 2535.2 RU. The chip was deactivated by injecting 1 M ethanolamine hydrochloride for 800 s at 10 μl/min into both channels.

For kinetics/affinity analysis, PTS was diluted in Running Buffer (1 × PBS-P, 5% DMSO) to seven concentrations (400, 200, 100, 50, 25, 12.5, and 0 μM), and injected into Fc1-Fc4 channels at 30 μl/min for a 90 s association phase, followed by a 120 s dissociation phase. A similar procedure was used for RES, diluted in Running Buffer to seven concentrations (400, 200, 100, 50, 25, 12.5, and 0 μM), with the same association and dissociation times. All measurements were carried out in Running Buffer at 30 μl/min flow rate.

### Prediction of transcription factor binding sites (TFBS) and electrophoretic mobility shift assay (EMSA)

The *Sirt1* promoter sequence of mouse (upstream 2 kb) was obtained from the National Center for Biotechnology Information. Potential ERα binding sites in the *Sirt1* promoter were predicted using hTFtarget, animalTFDS, and JASPAR. A common binding site with high scores predicted by all tools was selected for further EMSA probe design, and the core sequence was identified as GCCACGTGAC. HDOCK (a molecular docking server) was used to perform ERα-the core sequence of *Sirt1* promoter sequence docking.

Biotinylated SIRT1 probes (Bio-SIRT1), unlabeled competitive probes (Cold-SIRT1), and mutated unlabeled competitive probes (mut-SIRT1) were synthesized based on the core sequence (Table S4). DNA oligos were annealed using an Annealing Buffer for DNA Oligos (5 × ) (D0251, Beyotime) to form double-stranded DNA probes. EMSA was performed using the Chemiluminescent EMSA Kit (GS009, Beyotime) according to the manufacturer's protocol. Briefly, a 10 μl reaction mixture containing nuclease-free water, EMSA/Gel-Shift Binding Buffer, nuclear protein, and the probe was subjected to electrophoresis on a 6% polyacrylamide gel, followed by transfer to a nitrocellulose membrane using a wet Trans-Blot system. After transfer, crosslinking was performed using UV light to covalently bind the DNA–protein complexes to the membrane. Signals were detected using a ChemiDoc XRS Imager System (Bio-Rad).

### Plasmid construction and extraction

The plasmids for dual-luciferase reporter assays, including pcDNA3.1-basic, pcDNA3.1-ERα (containing ERα coding sequence), pGL3-basic, pGL3-SIRT1 (with 2-kb SIRT1 promoter), pGL3-mutSIRT1 (mutant SIRT1 promoter), and PRL-TK, were obtained from Genecreate and verified by sequencing. Additionally, HA-SIRT1 (with HA-tagged human SIRT1 coding sequences pcDNA3.1), Flag-ERα (with Flag-tagged human ERα coding sequences in pcDNA3.1), and Flag-mutERα (containing Flag-tagged human ERα coding sequences with the mutation of Pro325 to Ala and lysine-to-arginine substitutions at K8, K32, K171, K244, K252, K266, K268, K299, K302, K303, and K472) were provided by Genecreate and validated by sequencing. The EGFP-MDM2 plasmid was obtained from MiaoLingPlasmid. Plasmid extraction was performed using the Plasmid Maxi Preparation Kit (D0026, Beyotime).

### Dual-luciferase reporter assay

Plasmids including pcDNA3.1-basic, pCDNA3.1-ERα, pGL3-basic, pGL3-SIRT1, pGL3-mutSIRT1, and PRL-TK were co-transfected into 293T cells in different combinations for 6 h. After medium replacement, cells were incubated for an additional 24 h. Dual-luciferase reporter assays were performed using the Dual Luciferase Reporter Gene Assay Kit (11402ES80, Yeasen Biotechnology) according to the manufacturer's protocol.

### Statistical analysis

All statistical analyses were performed using SAS 9.4 software (SAS Institute Inc, Cary, NC, USA). Data distributions were assessed for normality and homogeneity of variance before analysis. For non-normally distributed data, the Kruskal-Wallis test was employed for comparisons. One-way ANOVA was used for multiple-group comparisons, followed by Duncan's multiple range test for *post hoc* analysis when appropriate. Comparisons between two groups were performed using Student’s *t* test or the Mann–Whitney U test for non-normally distributed data. Correlation analyses were conducted using Pearson's correlation coefficient. Graphical representations of the data were generated using GraphPad Prism 8.0 (GraphPad Software, Inc). All data are presented as mean ± standard deviation (SD).

## Data availability

All data are contained within the manuscript.

## Supporting information

This article contains supporting information (Figs. S1–S14 and Tables S1–S4).

## Conflict of interest

The authors declare that they have no conflicts of interest with the contents of this article.
