# Supplementary material for: ERα/SIRT1 signaling mediates the metabolic regulatory benefits of pterostilbene
Source: J Biol Chem. 2026 Aug 24;302(10):113471. doi: 10.1016/j.jbc.2026.113471 (PMC13599633; doi:10.1016/j.jbc.2026.113471)
Supplement: Supplementary file 1 [file mmc1.docx]

Supplementary materials for

**ERα/SIRT1 signaling mediates the metabolic regulatory benefits of pterostilbene**

Tengteng Huang ^1,§^, Xiaoling Chen ^1,§^, Daiwen Chen ^1^, Jingdong Yin ^2^, Bing Yu ^1^, Hui Yan ^1^, Ping Zheng ^1^, Zhiqing Huang ^1,*^

*^1^ Key Laboratory for Animal Disease-Resistance Nutrition of China Ministry of Education, Institute of Animal Nutrition, Sichuan Agricultural University, Chengdu, Sichuan 611130, P. R. China*

*^2^ State Key Laboratory of Animal Nutrition and Feeding, College of Animal Science and Technology, China Agricultural University, Beijing 100193, P. R. China*

^§^ These authors contributed equally to this work.

^*^ Correspondence: Zhiqing Huang, Institute of Animal Nutrition, Sichuan Agricultural University, Chengdu, Sichuan 611130, P. R. China; Tel/Fax: 86-28-86290922; E-mail: zqhuang@sicau.edu.cn.

**This file includes:**

Supplementary Fig. 1 to 14

Supplementary Tables 1 to 4

**Supplementary Fig. 1.** **Expression of lipid metabolism-related proteins in iWAT and mitochondrial complex proteins in BAT – related to Fig. 1**

(A) The protein expression levels of PPARγ, HSL, and p-HSL in iWAT.

(B) The protein expression of mitochondrial electron transport chain complexes I–V in BAT.

For all panels, n=3 per group. One-way ANOVA followed by Duncan's multiple range test was applied for HFD groups (HFD, HFD+RES, HFD+PTS) and chow diet groups (Control, RES, PTS). ^#^P < 0.1, *P < 0.05, **P < 0.01, and ***P < 0.001. Data are presented as means ± SD.

**Supplementary Fig. 2. RES and PTS enhance endurance capacity and promote** **skeletal muscle oxidative phenotype under standard diet and HFD – related to Fig. 1**

(A) Running distance and time in endurance test.

(B) Skeletal muscle weights and representative images of skeletal muscle morphology.

(C-D) The protein expression of slow MyHC and fast MyHC in TA (C) and GAS (D) muscle.

(E) The protein expression of mitochondrial complexes in SOL muscle.

(F) TEM images of mitochondria in SOL muscle, blue arrows mark ultrastructural alterations.

For (A), n=10 per group. For (B), n=20 per group. For (C)-(E), n=3 per group. One-way ANOVA followed by Duncan's multiple range test was applied for HFD groups (HFD, HFD+RES, HFD+PTS) and chow diet groups (Control, RES, PTS). Data are presented as means ± SD. *P < 0.05, ** P < 0.01, and ***P < 0.001.

**Supplementary Fig. 3. Mitochondrial complex protein expression and mitochondrial DNA copy number analysis in SOL and BAT – related to Fig. 2**

(A-B) Fasting and cold exposure body surface temperature.

(C-D) The protein expression of mitochondrial complexes (C) and mitochondrial DNA copy number (D) in SOL muscle.

(E-F) The protein expression of mitochondrial complexes (E) and mitochondrial DNA copy number (F) in BAT.

(G-H) The mRNA expression of thermogenic markers in BAT (G) and iWAT (H).

For (A)-(B), n=8-9 per group. For (C)-(F), n=3 per group. For (G)-(H), n=4 per group. One-way ANOVA followed by Duncan's multiple range test was applied for multiple-group comparisons. Asterisks directly above bars indicate significance compared to the control group. Horizontal lines with asterisks indicate direct comparisons between the connected groups. **P* < 0.05, ***P* < 0.01, and ****P* < 0.001. Data are presented as means ± SD.

**Supplementary Fig. 4. Effects of RES and PTS on muscle fiber type in C2C12 myotubes and lipid metabolism in 3T3-L1 adipocytes – related to Fig. 2**

(A) Differentiated C2C12 myotubes treated with DMSO control, RES or PTS for 1 day (panels below follow the same treatment in C2C12 myotubes). The protein expression of slow MyHC and fast MyHC.

(B) The mRNA expression of *Myh7*, *Myh2*, *Myh1*, and *Myh4* in C2C12 myotubes.

(C–D) The mRNA expression of genes encoding mitochondrial electron transport chain complexes (C) and transcription factors regulating mitochondrial biogenesis (D) in C2C12 myotubes.

(E) The protein expression of mitochondrial complexes in C2C12 myotubes.

(F) Differentiated 3T3-L1 adipocytes treated with DMSO control, RES or PTS for 1 day. Protein expression of FASN, UCP1, p-HSL, and total HSL.

For (B)-(E), n=4 per group. For (F), n=3 per group. One-way ANOVA followed by Duncan's multiple range test was performed for multiple-groups comparisons. Asterisks directly above bars indicate significance compared to the control group. Horizontal lines with asterisks indicate direct comparisons between the connected groups. **P* < 0.05, ***P* < 0.01, and ****P* < 0.001. Data are presented as means ± SD.

**Supplementary Fig. 5. Seahorse analysis in C2C12 myotubes– related to Fig. 2**

(A) Differentiated C2C12 myotubes were treated with DMSO control, 2 μM RES, or 2 μM pterostilbene for 1 day. The same treatment conditions were used for all panels. Oxygen consumption rate (OCR) was measured following sequential injection of oligomycin, FCCP, and rotenone/antimycin A (ROT/AA).

(B–E) Quantification of mitochondrial respiratory parameters, including basal respiration (B), maximal respiration (C), ATP production (D), and spare respiratory capacity (E).

(F) Extracellular acidification rate (ECAR) was measured following sequential injection of glucose, oligomycin, and 2-deoxy-D-glucose (2-DG).

(G–I) Quantification of glycolytic parameters, including glycolysis (G), glycolytic capacity (H), and glycolytic reserve (I).

n=3 per group. One-way ANOVA followed by Duncan’s multiple range test. ^#^*P* < 0.1, **P* < 0.05, ***P* < 0.01, and ****P* < 0.001. Data are presented as means ± SD.

**Supplementary Fig. 6. Virtual screening and protein interaction analysis of SIRT1 activators, RES, and PTS targets – related to Fig. 3**

(A) Predicted targets of SIRT1 activators using PharmMapper (<http://lilab-ecust.cn/pharmmapper/index.html>). Targets with intersection size > 5 are shown.

(B) Predicted targets of RES and PTS using PharmMapper. The top 10 target scores of RES and PTS are displayed.

(C) Venn diagram of the intersection between RES and PTS targets (Top 50) and SIRT1 activator targets (intersection size > 5).

(D) Protein-protein interaction (PPI) network analysis. PPI analysis was conducted using the STRING database (https://string-db.org/) with the confidence level set to highest confidence (default score > 0.9).

**Supplementary Fig. 7. mRNA expression profile of *Esr1* in mice – related to Fig. 3**

(A) The single-cell gene expression profile of *Esr1* was obtained from the Tabula Muris database (<https://tabula-muris.sf.czbiohub.org/>), a compendium of single cell transcriptome data from the mice, where blue dots represent fat and red dots represent skeletal muscle.

(B) *Esr1* mRNA levels across mouse tissues by qPCR, normalized to *Arf1* (n = 4 per group).

**Supplementary Fig. 8. 2D Structure of small molecules and RMSD Plot of small molecule-ERα in molecular dynamics (MD) simulation – related to Fig. 3**

(A) The 2D structure of the small molecules.

(B) The RMSD plot of small molecule-ERα in MD simulation.

**Supplementary Fig. 9.**  Surface plasmon resonance (SPR) analysis of the interactions between pterostilbene, E2, and ERα **– related to Fig. 3**

(A) SPR sensorgrams and steady-state binding analysis of E2–ERα interaction.

(B) SPR sensorgrams and steady-state binding analysis of PTS–ERα interaction.

(C) Competitive SPR assay of PTS and E2 binding to ERα. E2 and PTS were co-incubated at indicated concentrations before injection onto the ERα-immobilized sensor chip.

**Supplementary Fig. 10. Pro325 is the key amino acid for PTS-mediated ERα-SIRT1 interaction – related to Fig. 5**

(A and B) Preferred interacting amino acids of RES or PTS with the ERα-SIRT1 complex in molecular docking and free energy decomposition in molecular dynamics (MD) simulations.

(C) Structural model of the mutERα (proline 325 to alanine substitution).

(D and E) MD simulation of RES (D) or PTS (E) with mutERα-SIRT1. RMSD plots and binding free energies are shown.

(F and G) 293T cells transfected with HA-SIRT1 and Flag-ERα (or Flag-mutERα) were treated with 2 μM PTS for 1 day. Co-IP analysis of ERα-SIRT1 or mutERα-SIRT1 interaction (F). Co-IP was also performed to detect Ub-ERα and Ub-SIRT1 expression (G).

(H) 293T cells transfected with HA-SIRT1 and Flag-ERα (or Flag-mutERα) were treated with 2 μM PTS for 19 hours, followed by treatment with CHX (50 μg/mL) + 2 μM PTS for 5 hours. Proteins were collected every hour for 5 hours, and Western blotting was used to detect ERα and SIRT1 protein expression.

**Supplementary Fig. 11. Molecular dynamics (MD) simulations of ERα or SIRT1 with MDM2 – related to Fig. 6**

The RMSD curves and protein conformations are shown for the simulation period of 0-50 ns in MD simulation.

**Supplementary Fig. 12. Effects of skeletal muscle-specific ERα knockout on the PTS-promoted skeletal muscle oxidative phenotype – related to Fig. 8**

(A) The mRNA expression of *Esr1* and *Sirt1* in TA muscle.

(B) The protein expression of ERα and SIRT1 in TA muscle.

(C) Representative images of TA muscle morphology.

(D) The mRNA expression of *Myh7*, *Myh2*, *Myh1*, and *Myh4* in TA muscle.

(E) The protein expression of slow MyHC and fast MyHC in TA muscle.

(F) The protein expression of mitochondrial complexes in SOL muscle.

For (A) and (D), n=7 per group in Con f/f group and Con f/f + PTS group, n=8 per group in MERKO + PTS group. For (B), (E), and (F), n=4 per group. One-way ANOVA followed by Duncan’s multiple-range test was performed for multiple-group comparisons. **P* < 0.05, ***P* < 0.01, and ****P* < 0.001. Data are presented as means ± SD.

**Supplementary Fig. 13. Effects of skeletal muscle-specific ERα knockout on PTS-improved metabolic health in female mice – related to Fig. 8.**

(A) Body weight gain curves during the 13-week experimental period.

(B) Body weight gain at the end of the experiment and representative photographs of mice selected based on average body weight.

(C) Weights of BAT, iWAT, and gWAT, along with representative images.

(D) Lean and fat composition measured by body composition analysis.

(E) Fasting and cold exposure body surface temperature.

(F) Grip strength measured by grip strength test.

(G) Running distance in endurance test.

(H) Respiratory exchange ratio (RER), average hourly oxygen consumption (VO_2_), and VO_2_ over time curves.

(I and J) Blood glucose levels at different time points and corresponding area under the curve (AUC) in GTT (I) and ITT (J).

For all panels, n=7 per group in Con f/f group and Con f/f + PTS group and n=8 per group in MERKO + PTS group. One-way ANOVA followed by Duncan’s multiple range test. ^#^*P* < 0.1, **P* < 0.05, and ***P* < 0.01. Data are presented as means ± SD.

**Supplementary Fig. 14. PTS promotes skeletal muscle oxidative phenotype in C2C12 myotubes via the ERα/SIRT1 signaling pathway – related to Fig. 8**

(A) Differentiated C2C12 myotubes treated with DMSO control, 2 μM PTS, 2 μM PTS + 5 μM ERα antagonist (ICI 182780), or 2 μM PTS + 5 μM SIRT1 inhibitor (EX527) for 1 day (panels below follow the same treatment in C2C12 myotubes). The protein expression of ERα, SIRT1, p-AMPK, and AMPK.

(B) The protein expression of slow MyHC and fast MyHC in C2C12 myotubes.

(C) The mRNA expression of *Myh7*, *Myh2*, *Myh1*, and *Myh4* in C2C12 myotubes.

(D) Representative immunofluorescence images of slow MyHC (green fluorescence) in C2C12 myotubes. Magnification: ×100.

(E) Representative images of live-cell mitochondrial staining (red fluorescence) in C2C12 myotubes. Magnification: ×100.

(F) The protein expression of mitochondrial complexes in C2C12 myotubes.

(G) Differentiated C2C12 myotubes were transfected with control siRNA (si-NC) or siRNA targeting ERα (si-ERα), followed by treatment with DMSO or PTS for 1 day. The protein expression levels of ERα, SIRT1, slow MyHC, and fast MyHC were detected in C2C12 myotubes.

For (F), n=4 per group. For all other panels, n=3 per group. One-way ANOVA followed by Duncan’s multiple-range test was performed for multiple-group comparisons. ^#^P < 0.1, *P < 0.05, **P < 0.01, and ***P < 0.001. Data are presented as means ± SD.

**Supplementary Table 1. The SIRT1 activator library**

| **SIRT-1 activators** | **CAS** |
| --- | --- |
| 3β,6α,12β-Dammar-E-20(22)-ene-3,6,12,25-tetraol | 97744-95-1 |
| Agrimol B | 55576-66-4 |
| Ainsliadimer C | 1365995-73-8 |
| Butein | 487-52-5 |
| CAY10602 | 374922-43-7 |
| cis-RES | 61434-67-1 |
| E1231 | 1031195-19-3 |
| KL1333 | 1800405-30-4 |
| Nicotinamide riboside | 1341-23-7 |
| Ophiopogonin D' | 65604-80-0 |
| Quercetin | 117-39-5 |
| RES | 501-36-0 |
| RES-d4 | 1089051-56-8 |
| Scopolin | 531-44-2 |
| SIRT1 activator 3 | 839699-72-8 |
| SIRT1-A2 | 304448-98-4 |
| Sirtuin modulator 5 | 694469-31-3 |
| SRT 1460 | 925432-73-1 |
| SRT 2104 | 1093403-33-8 |
| SRT 2183 | 1001908-89-9 |
| SRT1720 HCl | 1001645-58-4 |
| SRT3025 HCl | 2070015-26-6 |
| SRTCX1002 | 1203479-63-3 |
| SRTCX1003 | 1203480-86-7 |

**Supplementary Table 2. Binding free energy of ligands as ERα-SIRT1 PPI stabilizers to ERα or SIRT1 in MD simulations**

| **Ligand** | **Binding free energy in MD mimic (KJ/mol)** | | | |
| --- | --- | --- | --- | --- |
|  | **G_Complex_** | **G_ERα_** | **G_SIRT1_** | **ΔG** |
| Cis-RES | -25.05 | -31.583 | -31.317 | 0.266 |
| RES | -32.693 | -12.492 | -11.909 | 0.583 |
| PTS | -79.182 | -17.597 | -9.097 | 8.5 |
| Butein | -30.952 | -35.612 | -48.894 | 13.282 |
| SRT1460 | -45.275 | -35.438 | -48.815 | 13.377 |
| Sirt1 activator 3 | -86.085 | -41.341 | -26 | 15.341 |
| Genistein | -17.556 | -44.615 | -17.447 | 27.168 |
| SRTC1002 | -87.928 | -41.089 | -69.806 | 28.717 |
| E2 | -70.209 | -6.606 | -36.672 | 30.066 |
| Sirt1 M5 | -66.703 | -28.697 | -66.347 | 37.65 |
| Quercetin | -5.535 | -66.305 | -11.427 | 54.878 |
| trans-RES-d4 | -20.043 | -57.398 | -0.756 | 56.642 |
| E1231 | -64.645 | -83.371 | -17.557 | 65.814 |
| KL1333 | -38.788 | -84.53 | -10.381 | 74.149 |
| Sirt1 M1 | -83.254 | -144.587 | -46.472 | 98.115 |

G_Complex_: Binding free energy between the ligand and the ERα-SIRT1 complex, G_ERα_: Binding free energy between the ligand and ERα, G_SIRT1_: Binding free energy between the ligand and SIRT1. ΔG=|G_ERα_-G_SIRT1_|.

**Supplementary Table 3. Primers used for quantitative PCR (qPCR)**

| **Gene name** | **Primer Sequence (5'-3')** | | **GeneBank ID** | **Product size** |
| --- | --- | --- | --- | --- |
| *Myh7* | Forward | CTTCTACAGGCCTGGGCTTAC | NM_080728 | 128 |
|  | Reverse | CTCCTTCTCAGACTTCCGCAG |  |  |
| *Myh2* | Forward | TTCCAGAAGCCTAAGGTGGTC | NM_001039545 | 94 |
|  | Reverse | GCCAGCCAGTGATGTTGTAAT |  |  |
| *Myh1* | Forward | CAACCCATACGACTACGCCT | NM_030679 | 119 |
|  | Reverse | CATCAGAAGTGAAGCCCAGAAT |  |  |
| *Myh4* | Forward | CTTGTCTGACTCAAGCCTGCC | NM_010855 | 158 |
|  | Reverse | TCGCTCCTTTTCAGACTTCCG |  |  |
| *Nrf1* | Forward | TGCTTCAGAACTGCCAACCA | [NM_001410232.1](https://www.ncbi.nlm.nih.gov/entrez/viewer.fcgi?db=nucleotide&id=-2013996468) | 105 |
|  | Reverse | ATTTCACCGCCCTGTAACGT |  |  |
| *Nrf2* | Forward | AAAGCACAGCCAGCACATTC | NM_010902 | 86 |
|  | Reverse | TGGGATTCACGCATAGGAGC |  |  |
| *Polrmt* | Forward | TTGACCAGCAGAAGCAAGCC | [NM_001407795.1](https://www.ncbi.nlm.nih.gov/entrez/viewer.fcgi?db=nucleotide&id=-2044949888) | 135 |
|  | Reverse | GCTGCTTTTCCTCTGAGTTCGT |  |  |
| *Tfam* | Forward | ATTTCACCGCCCTGTAACGT | NM_009360 | 193 |
|  | Reverse | TCGTTTCACACTTCGACGGAT |  |  |
| *Tfb1m* | Forward | GAAGCACAACGCCTCGAAAG | NM_146074 | 91 |
|  | Reverse | AATGCATGAGCGAGAGGTGG |  |  |
| *Tfb2m* | Forward | CCAATAATACGCCATTTACGTT | NM_001331055.1 | 178 |
|  | Reverse | GGCAGTAGTCATAGATCCAC |  |  |
| *Ndufa1* | Forward | GTCAATCGCTACTATGTGTCC | NM_019443.2 | 117 |
|  | Reverse | TGCATAGCCTTCTAACAGGA |  |  |
| *Ndufb3* | Forward | GCAACATCACCTTCCCGAGT | [NM_025597.3](https://www.ncbi.nlm.nih.gov/entrez/viewer.fcgi?db=nucleotide&id=922959870) | 70 |
|  | Reverse | AAAGCTACCACAAACGCAGCA |  |  |
| *Sdha* | Forward | CCACTCACTCTTACACACGTT | [NM_023281.1](https://www.ncbi.nlm.nih.gov/entrez/viewer.fcgi?db=nucleotide&id=54607097) | 89 |
|  | Reverse | CCATCAGAAGATCCAGTGCAA |  |  |
| *Sdhb* | Forward | ACCCCTTCTCTGTCTACCG | [NM_001355515.1](https://www.ncbi.nlm.nih.gov/entrez/viewer.fcgi?db=nucleotide&id=1243457569) | 130 |
|  | Reverse | AATGCTCGCTTCTCCTTGTAG |  |  |
| *Uqcrb* | Forward | TGCCTCATAGTCAGGTCCA | [NM_026219.2](https://www.ncbi.nlm.nih.gov/entrez/viewer.fcgi?db=nucleotide&id=1377224727) | 126 |
|  | Reverse | GTTAATGCGAGATGATACACT |  |  |
| *Uqcrq* | Forward | CTGATCTACACATGGGGCAAC | [NM_025352.3](https://www.ncbi.nlm.nih.gov/entrez/viewer.fcgi?db=nucleotide&id=1031698820) | 56 |
|  | Reverse | GCTGGATTCTTCCTTTTCGACT |  |  |
| *Cox5b* | Forward | ACCCTAATCTAGTCCCGTCC | NM_009942.2 | 89 |
|  | Reverse | CAGCCAAAACCAGATGACAG |  |  |
| *Cox6a1* | Forward | CATCAGGACCAAGCCCTTC | [NM_007748.5](https://www.ncbi.nlm.nih.gov/entrez/viewer.fcgi?db=nucleotide&id=1780282690) | 95 |
|  | Reverse | ACTCATCTTCATAGCCGGTCG |  |  |
| *Atp5a1* | Forward | CATTGGTGATGGTATTGCGC | [NM_007505.2](https://www.ncbi.nlm.nih.gov/entrez/viewer.fcgi?db=nucleotide&id=126506325) | 134 |
|  | Reverse | TCCCAAACACGACAACTCC |  |  |
| *Atp5b* | Forward | GAGGGCAATGATTTATACCAT | [NM_016774.3](https://www.ncbi.nlm.nih.gov/entrez/viewer.fcgi?db=nucleotide&id=133892441) | 92 |
|  | Reverse | ATCTGTCCATATACCAACGCTA |  |  |
| *Rplp0* | Forward | ACAGATTTGTCAAGCGCATC | [NM_007475.5](https://www.ncbi.nlm.nih.gov/entrez/viewer.fcgi?db=nucleotide&id=254939638) | 76 |
|  | Reverse | TTCCACAGGCCGATAGTAAGCA |  |  |
| *mtND1* | Forward | AATCGCCATAGCCTTCCTAA | NC_005089.1 | 114 |
|  | Reverse | GCGTCTGCAAATGGTTGTAA |  |  |
| CD36 | Forward | TGTGATCGGAACTGTGGGC | XM_030254088.1 | 80 |
|  | Reverse | ACTGGCATGAGAATGCCTCC |  |  |
| CD137 | Forward | AGTTTTGCTCCTCTACCCACA | NM_001077509.1 | 177 |
|  | Reverse | GACAGACGCCAGTACCGTTC |  |  |
| Cidea | Forward | AGCAACCAAAGAAATCGGGAA | NM_007702.2 | 108 |
|  | Reverse | ACATCTCGTACATCGTGGCTT |  |  |
| Dio2 | Forward | GTGAAGATGCTCCCAATTCCA | NM_010050.4 | 69 |
|  | Reverse | CCGAGGCATAATTGTTACCTG |  |  |
| TBX1 | Forward | GAAACTGACCAATAACCTGCT | NM_011532.2 | 114 |
|  | Reverse | TTCTCACTGTCTTTTCGAGGG |  |  |
| TMEM26 | Forward | GCACCATCACTAGAGACCAAC | NM_177794.3 | 53 |
|  | Reverse | CGTCCCCACAAACATCAGA |  |  |
| *Esr1* | Forward | AAGAATAGCCCTGCCTTGTCC | NM_001302533.1 | 58 |
|  | Reverse | CATCCAACAAGGCACTGACCA |  |  |
| *Esr1*  (exon 3) | Forward | GCCAGAATGGCCGAGAGAG | NM_001302533.1 | 123 |
|  | Reverse | CCCCATAATGGTAGCCAGAGG |  |  |
| *ESR1*  (Human) | Forward | AGTGTGCAATGACTATGCTT | NM_001385572.1 | 222 |
|  | Reverse | CTTCGGTCTTTTCGTATCCCA |  |  |
| *Sirt1* | Forward | ACAGTGACAGTGGCACATGC | NM_019812 | 130 |
|  | Reverse | AATCCAGATCCTCCAGCACA |  |  |
| *SIRT1*  (Human) | Forward | AAGGAAAACTACTTCGCAAC | NM_001142498.2 | 156 |
|  | Reverse | AAATATCTCCTCGTACAGCTT |  |  |
| *Arf1* | Forward | CGCCACTACTTCCAGAACACC | NM_007476.4 | 65 |
|  | Reverse | ACACGCTCTCTGTCATTGCT |  |  |
| *Gapdh* | Forward | AGGGCATCTTGGGCTACAC | NM_008084 | 211 |
|  | Reverse | TGGTCCAGGGTTTCTTACTCC |  |  |
| *GAPDH* (Human) | Forward | TCTGACTTCAACAGCGACACC | NM_002046.7 | 117 |
|  | Reverse | GTTGCTGTAGCCAAATTCGTT |  |  |

**Supplementary Table 4. The probe sequence of Sirt1 in ERα-Sirt1 EMSA assay**

| **Probe name** | **Sequence** |
| --- | --- |
| Bio-Sirt1 | Biotin-5'-TACGCCCGGCCACGTGACCCGGCGTT-3'-Biotin  Biotin-3'-ATGGGCCGGTGCACTGGGCCGCAA-5'-Biotin |
| Cold-Sirt1 | 5'-TACGCCCGGCCACGTGACCCGGCGTT-3'  3'-ATGCGGGCCGGTGCACTGGGCCGCAA-5' |
| Mut-Sirt1 | 5'-TACGCCCGATTGTACAGTCCGGCGTT-3'  3'-ATGCGGGCTAACATGTCAGGCCGCAA-5' |

The underline represents the predicted binding site of *Sirt1*, bio-Sirt1 means oligonucleotide probes that labeled with biotin, cold-Sirt1 means oligonucleotide probes that did not label with biotin, mut-Sirt1 means oligonucleotide probes that were mutated at the binding site.
